# Toward Sustainable Lagoon Wastewater Treatment: A Review of Nutrient Management Technologies and Their Suitability for Small Communities

**DOI:** 10.1021/acsestwater.5c00757

**Published:** 2025-10-02

**Authors:** Denis S. Ruto, Ziya S. Jang, Pablo K. Cornejo, Harold L. Leverenz, Kevin D. Orner

**Affiliations:** † Wadsworth Department of Civil and Environmental Engineering, 5631 West Virginia University, 1306 Evansdale Dr, PO Box 6103, Morgantown, West Virginia 26506-6103, United States; ‡ Department of Civil and Environmental Engineering, 8789 University of California, Davis, One Shields Ave., Davis, California 95616-5270, United States; § Department of Civil and Environmental Engineering, 14663 California State University, Chico, 400 West First Street, Chico, California 95929, United States

## Abstract

Lagoon wastewater systems are popular in small communities (<10,000 people) due to their cost-effectiveness and energy efficiency. However, these systems struggle to meet regulatory discharge limits for ammonia, total nitrogen, and total phosphorus, emphasizing the need for improved nutrient management. Most existing reviews focus on large-scale mechanical systems, leaving a gap for stand-alone lagoon systems in resource-limited settings. This systematic review analyzed 1003 peer-reviewed articles spanning five decades, evaluating nutrient management technologies for municipal lagoons. Technologies were categorized by nutrient target, process type, installation location, and development phase. Biological processes dominate (79%) due to their adaptability and cost-effectiveness, while advanced and hybrid systems are gaining traction. Performance varied widely based on design, climate, and operational conditions, highlighting the importance of site-specific considerations. To support context-sensitive selection, this study developed the Suitability Index (SIDX), a multicriteria framework incorporating complexity, automation, availability, and operational feasibility. SIDX identified several applicable and promising technologies for small-community lagoons. The review also highlighted an evolving focus toward circularity, resource recovery, and emissions reduction, moving beyond traditional pollutant removal. These insights provide practical guidance to support adaptive, context-appropriate nutrient management strategies aligned with current regulatory standards and future environmental goals.

## Graphical abstract

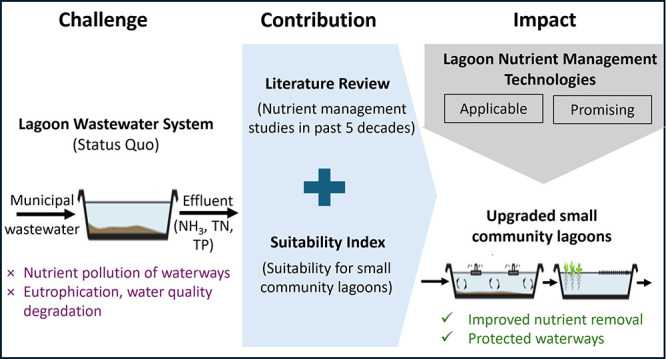

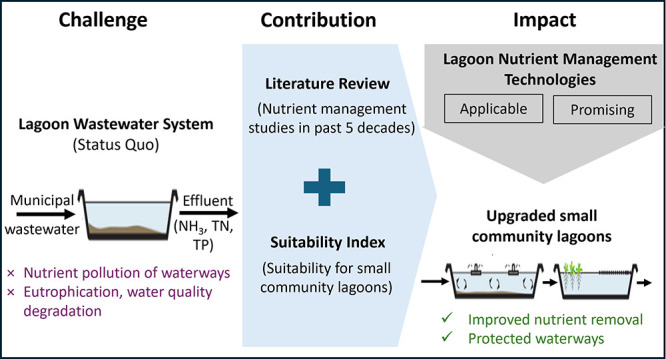

## 1 Introduction

Lagoon wastewater treatment systems, also known as waste stabilization ponds, are engineered shallow basins designed to treat wastewater through natural biological and physical processes. Lagoon systems are generally categorized into three types: anaerobic, facultative, and aerated, and may operate as stand-alone units or as components of hybrid systems.^1,2^ For municipal applications, the most common configurations are stand-alone facultative or aerated lagoons.^3^ Over 8,000 lagoon systems are currently in operation across the United States, primarily serving small communities with populations under 10,000.^4^ Their widespread use is largely attributed to their low energy demands, operational simplicity, and long-term cost-effectiveness.^5^ However, while facultative lagoon systems typically achieve 75–85% removal of BOD and 70–87% of TSS, nitrogen and phosphorus removal is generally limited to below 50 and 35%, respectively, levels that often fall short of current effluent quality standards (Table 1).

**Table 1:** Performance of Typical Lagoon Wastewater Systems

parameter	removal efficiency (%)	primary removal mechanism	references
biochemical oxygen demand	75–85	biological degradation	^1–3,6–8^
total suspended solids	70–87	sedimentation and filtration	^1–3,8^
ammonia	30–65	algal uptake and settling	^1,2,9,10^
nitrogen	<50	algal uptake and settling	^1,2,9–13^
phosphorus	<35	adsorption, algal-uptake, and settling	^1,2,14–17^

In the United States, discharging lagoon systems are regulated under the National Pollutant Discharge Elimination System (NPDES), a program established by the Clean Water Act to control point source pollution and protect water quality. NPDES permits define allowable pollutant discharge limits and require regular monitoring and reporting.^18^ However, the application of NPDES to lagoon systems reveals significant inconsistencies in both permitting and compliance. As illustrated in Figure 1A, U.S. lagoons have significantly more permits regulating ammonia discharge compared to total nitrogen (TN) and total phosphorus (TP), which are typically monitored without enforceable discharge limits. This disparity is further highlighted in Figure 1B, showing ammonia-specific discharge limits in 80% of lagoon permits, whereas TN and TP limits are present in only 4 and 12%, respectively.

**Figure 1:**
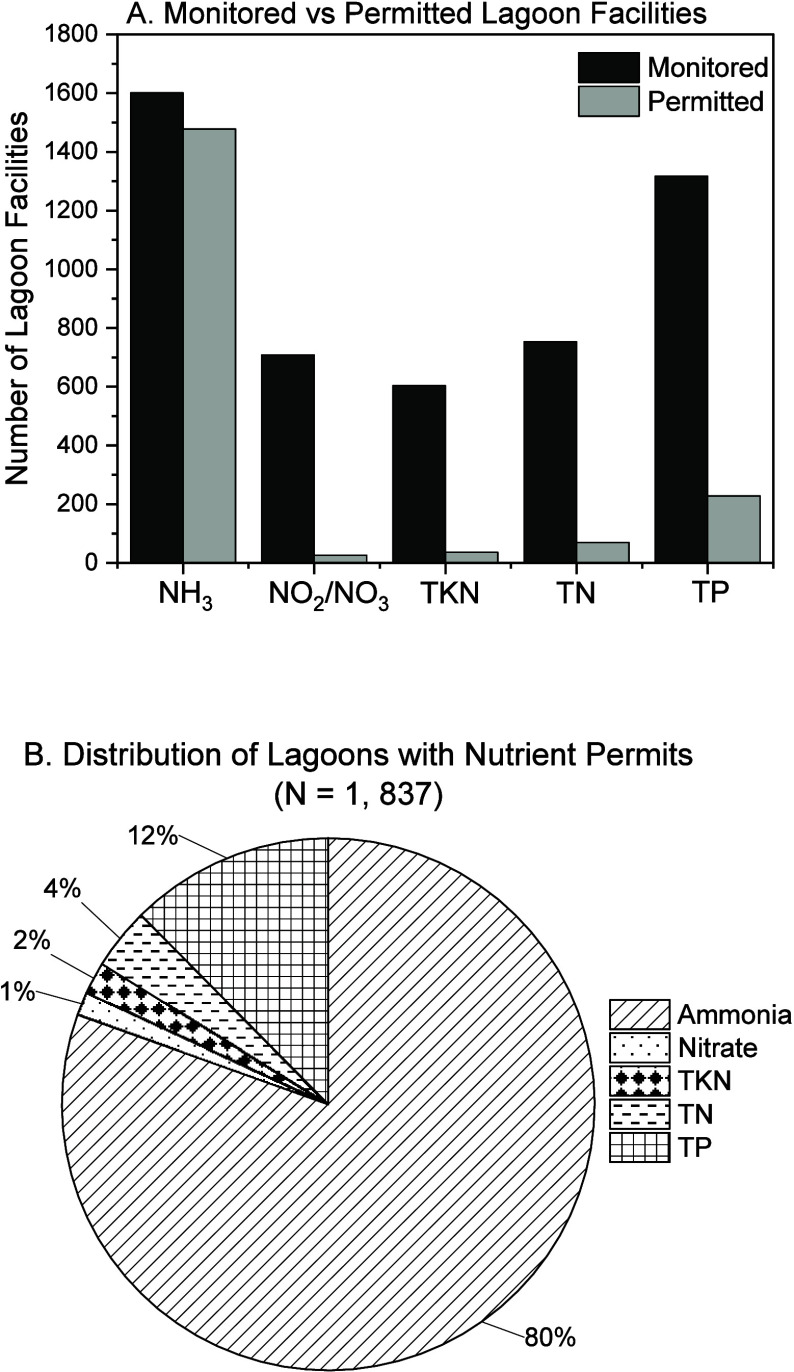
(A, B) Regulatory focus on nutrient parameters in lagoon wastewater systems across the United States as of July 2024. ‘Permitted’ indicates parameters with established limits that require periodic measurement and reporting. ‘Monitored’ refers to parameters tracked for informational purposes only. NH_3_: ammonia, NO_3_: nitrate; NO_2_: nitrite; TKN: total Kjeldahl nitrogen, TN: total nitrogen, and TP: total phosphorus.

A 2022 U.S. EPA assessment of 4657 discharging lagoons found that 61% exceeded effluent limits for at least one pollutant.^4^ Ammonia was the most frequently violated nutrient parameter, with 21% of systems exceeding regulatory thresholds. Although TN and TP are also key contributors to nutrient pollution, their limited inclusion in permits makes it difficult to assess compliance or drive meaningful management. This mismatch between nutrient-related violations and permitting practices highlights a critical regulatory gap and underscores the urgent need for more consistent, enforceable nutrient management strategies in lagoon systems.

Excess nutrients in wastewater present a national grand challenge in environmental engineering, significantly impacting public health and ecosystem stability.^19^ Ammonia is toxic to aquatic life and contributes to oxygen depletion in water bodies.^20^ Elevated nitrate concentrations in groundwater poses risks to drinking water safety, increasing the risk of methemoglobinemia in infants.^21^ Excess nitrogen and phosphorus accelerate eutrophication, causing algal blooms, hypoxia, and the formation of aquatic “dead zones,” severely disrupting ecosystems.^11,16,17^ Given the low nutrient removal rates of lagoons and limited TN and TP permitting, improving nutrient control in these systems is urgent.

Although nutrient removal technologies are well-studied, most reviews focus on large mechanical systems with flows greater than 1 million gallons/day. Municipal lagoon wastewater systems are rarely the primary focus with existing literature often treating them as preliminary steps paired with other technologies.^22–24^ Several reviews have categorized technologies based on nutrient type, emphasizing either nitrogen or phosphorus removal.^17,21,25,26^ Recent research emphasizes integrated nutrient removal and recovery.^27,28^ Other studies emphasize innovative solutions such as struvite precipitation, algal cultivation and active filters from other waste-streams.^16,29–31^ However, none comprehensively assess lagoon-specific nutrient strategies or evaluate them in the context of small communities that face significant resource constraints, including limited finances and technical expertise. Thus, identifying effective, practical, and affordable nutrient management strategies explicitly tailored to lagoon systems is needed.

Hydraulics also play a pivotal role in lagoon performance. Factors such as baffling, short-circuiting, inlet and outlet placement, mixing patterns, and sludge accumulation can strongly influence treatment outcomes, sometimes overshadowing the benefits of nutrient removal technologies themselves.^3^ For instance, poor hydraulics may reduce effective retention time and undermine biological treatment processes, while well-designed baffling can enhance contact between wastewater and microbial communities.^32^ Likewise, sludge accumulation can in some cases diminish effective volume, making periodic desludging important for sustaining treatment efficiency.^1,2^ Although hydraulics are not nutrient removal mechanisms in themselves, they represent a foundational determinant of lagoon effectiveness and should be considered alongside biological and chemical treatment processes.

This study addresses these gaps by systematically reviewing nutrient management strategies specifically for municipal lagoon wastewater systems in small communities. Unlike previous reviews, this analysis emphasizes lagoons’ unique operational characteristics, actual nutrient removal performance, and applicability to small-scale settings. We reviewed 1003 peer-reviewed articles, categorized relevant technologies, and evaluated their suitability for small-community lagoons. While contaminants like TSS, BOD, COD, and emerging pollutants are important, this review prioritizes nitrogen and phosphorus due to their central role in regulatory compliance and eutrophication concerns in lagoon systems.^1,3,33^

The paper outlines our systematic review methodology and presents a detailed discussion of ammonia, TN, and TP removal technologies, covering conventional, advanced, and hybrid approaches. A unique feature is the development of a Suitability Index (SIDX) to evaluate the feasibility of identified technologies for small community lagoon systems based on performance and operational criteria. The motivation for this work is further supported by the U.S. EPA’s *Lagoon Wastewater Treatment Action Plan (2022–2026)*, which prioritizes support for small, rural, and tribal communities relying on lagoon systems.^34^ This study supports that vision by providing the technical information needed to guide effective and equitable nutrient management in stand-alone municipal lagoon systems. Ultimately, this review aims to serve as a practical resource for lagoon operators, utility managers, and community stakeholders, enabling informed, context-specific decisions for nutrient management in small-community lagoon systems.

## 2 Methods

### 2.1 Scope and Literature Search

The primary objective of this literature review was to explore nutrient management strategies applicable to municipal lagoon wastewater systems, particularly for small communities with populations under 10,000. The scope of the review was intentionally limited to peer-reviewed literature examining nutrient management, either through removal or recovery, of nitrogen (including ammonia) and phosphorus. A systematic search strategy was employed across multiple academic databases, including the Civil Engineering Database, Web of Science, Compendex, Scopus, ScienceDirect, SciTech Connect, and Google Scholar. Since the shift toward nutrient management gained momentum in the late 20th century, driven by the Water Pollution Control Act of 1965 and the Clean Water Act of 1972, this review focused on studies published between 1968 and June 2024 to capture the evolution of nutrient management practices in lagoons.18 The literature search utilized specific keywords and phrases to capture studies on nutrient management which included: “ammonia removal”, “nitrogen removal”, “phosphorus removal”, “nutrient removal”, “nutrient recovery”, “domestic wastewater”, “lagoon wastewater”, and “waste stabilization ponds”. These broad search terms were selected to ensure the comprehensive inclusion of studies addressing various nutrient management approaches relevant to small-scale wastewater treatment.

### 2.2 Systematic Review Process Using the PRISMA Framework

The Preferred Reporting Items for Systematic Reviews and Meta-Analyses (PRISMA) tool was adapted to structure this systematic review, providing a transparent, rigorous approach to the selection, screening, and synthesis of relevant studies.^35^ PRISMA’s three step framework: (1) identification, (2) screening, and (3) eligibility and inclusion, was applied to ensure clarity and reproducibility throughout the review process.^36^

#### 2.2.1 Identification

Based on this review’s objective, a comprehensive search was conducted using the keywords detailed in Section 2.1 to retrieve a broad set of studies relevant to the objectives. Duplicate records from multiple databases were removed in this initial phase to ensure unique, relevant entries.

#### 2.2.2 Screening Process

A two-step screening process was followed to ensure accuracy in selecting relevant studies. First, titles and abstracts were screened to eliminate irrelevant studies, followed by a full-text review to further refine the selection based on predefined inclusion and exclusion criteria. Abstracts were reviewed in batches of 50 search results. Included studies focused on municipal wastewater treatment with an emphasis on nutrient management, covering either nutrient removal or recovery. Each technology needed to demonstrate suitability for small wastewater systems, with a preference for, but not limited to, lagoon wastewater systems. Studies focusing on industrial or agricultural wastewater, as well as technologies designed exclusively for large mechanical systems, were omitted due to their limited applicability to lagoon systems. This filtering process ensured that the studies retained were directly relevant to the nutrient management challenges faced by small communities using lagoon wastewater treatment systems. The entire screening process was documented in a PRISMA flow diagram to illustrate the number of articles retrieved, filtered, and included in the final analysis (Figure 2).

**Figure 2:**
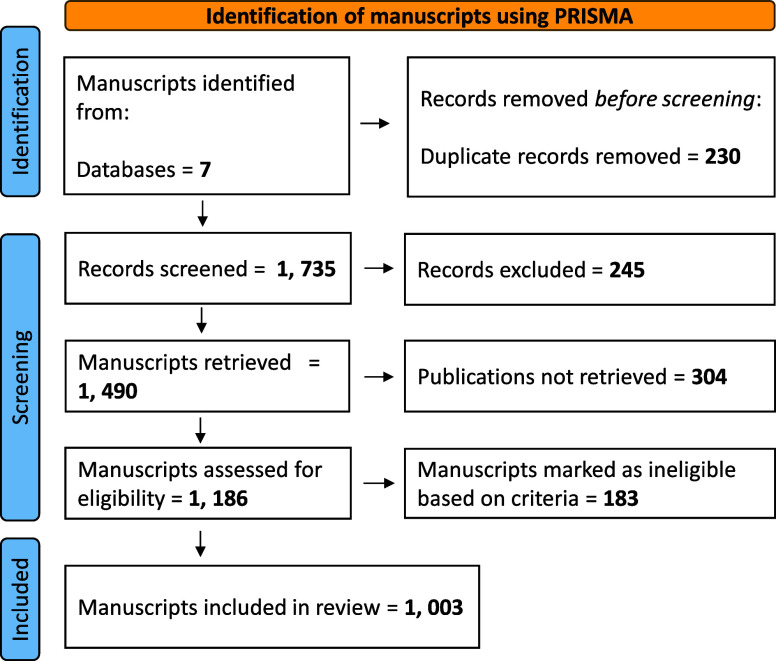
PRISMA flow diagram of reviewing studies on nutrient management in small municipal wastewater systems.

#### 2.2.3 Eligibility and Inclusion

Following the screening, eligible studies that met all criteria were included in the review. Data were systematically extracted from each study, focusing on specific metrics to maintain consistency in evaluation. These metrics included nutrient management technology, development phase (bench-scale, pilot-scale, or full-scale), nutrient removal efficiency (for ammonia, TN, and TP), underlying mechanisms, and installation characteristics (prelagoon, in-lagoon or add-on/postlagoon). Factors such as reliability of nutrient measurement methods, representativeness of lagoon wastewater conditions, and applicability to lagoon systems were also considered. This approach enabled a structured synthesis of findings, allowing for a comparative analysis of different nutrient management strategies.

### 2.3 Categorization and Presentation of Results

The nutrient management strategies were systematically organized into five categories: (i) process classification (biological, physical, chemical, or hybrid processes); (ii) type of nutrient removed (ammonia, TN, and TP), and removal mechanisms; (iii) underlying technology; (iv) installation location (prelagoon, in-lagoon or add-on/postlagoon); and (v) development phase (bench-scale, pilot-scale, or full-scale). This categorization allowed for detailed analysis and focused comparisons, enhancing understanding of the technologies’ strengths, limitations, and relevance to small-scale lagoon systems.

#### 2.3.1 Process Classification

Technologies were grouped by their dominant nutrient removal processes: biological, physical, chemical, or hybrid. Biological methods, such as nitrification-denitrification, use microbes to assimilate or convert nutrients.^37^ Physical processes involve sedimentation or filtration to separate nutrients from wastewater while chemical approaches include precipitation and coagulation using agents like alum or ferric salts.^38,39^ Hybrid systems combine multiple mechanisms, for example, integrating biological nitrogen removal with chemical phosphorus precipitation for broader nutrient control.^40^ This categorization facilitated comparisons within and across categories, such as evaluating biological versus physical systems or comparing different chemical precipitation methods, providing insights into each process’s strengths, limitations, and suitability for lagoon systems. It enabled comparative analysis, highlighting the trade-offs in complexity and effectiveness across different approaches.^41^

#### 2.3.2 Type of Nutrients and Removal Mechanism

Municipal lagoon influent typically contains three primary nutrient pollutants: ammonia, TN, and TP.^2^ These contribute to eutrophication and are subject to regulatory scrutiny.^33^ Ammonia originates from protein breakdown; TN includes ammonia, nitrate, nitrite, and organic nitrogen forms; and TP encompasses both dissolved and particulate phosphorus species.^2,42,43^

Removal occurs through natural lagoon processes such as algal and microbial uptake, and sedimentation, though often at lower removal efficiencies compared to advanced treatment systems.^3^ Biological nitrification-denitrification is the main pathway for nitrogen removal, where specific bacteria oxidize ammonia to nitrate under aerobic conditions and subsequently reduce nitrate to nitrogen gas under anoxic conditions.^11,44^ Other physical-chemical approaches such as ion exchange and advanced oxidation processes can also reduce nitrogen concentrations.^9^

Phosphorus removal typically relies on chemical precipitation using ferric or aluminum salts to form insoluble compounds that settle out.^45^ Alternatively, enhanced biological phosphorus removal (EBPR) uses specialized microbes to store phosphorus intracellularly, reducing the need for chemical inputs, although this process is sensitive to operational conditions.^46^ Adsorptive media such as biochar or industrial byproducts may also be employed to bind phosphorus through surface interactions.^14^

Classifying treatment strategies according to their underlying removal mechanisms provides a structured framework for assessing their relevance and effectiveness in lagoon contexts.^23^ Each mechanism presents unique trade-offs; for instance, while nitrification-denitrification offers high nitrogen removal efficiency, it demands precise control of oxygen and carbon levels, whereas chemical precipitation is robust but generates additional sludge.^47,48^ Emphasizing these core processes over commercial claims can enable communities to make more informed decisions, aligning technology selection with site-specific factors such as climate conditions, resource availability, and regulatory objectives.^41^

Additionally, this mechanism-based perspective also highlights opportunities for integrated solutions. For instance, pairing lagoon nitrification with packed-bed reactors can promote downstream denitrification.^49^ While wetlands alone offer limited phosphorus removal, their performance can be improved with engineered media or metal salt additions to reduce phosphorus release.^14,39^ Understanding these processes is essential to guide technology selection, avoid misaligned investments, and ensure cost-effective, sustainable nutrient management in small community lagoon systems.^45,50^

#### 2.3.3 Underlying Technologies

While nutrient management technologies may differ in design or branding, many share common foundational processes. For example, biological nutrient removal (BNR) systems may differ in their reactor configurations, aeration strategies, or bacterial community engineering, but the underlying nitrification-denitrification process is the same.^21,23,37^ Similarly, phosphorus removal technologies may vary in their choice of precipitation agents or their integration with filtration systems, yet the chemical basis of phosphorus removal is fundamentally unchanged.^46,47,51^

These commonalities in underlying processes allow for a wide range of solutions to be adapted for lagoon systems, from in-lagoon aeration systems designed to enhance nitrification to postlagoon add-ons like filtration beds for phosphorus polishing. This diversity of commercial solutions offers operators and decision-makers the flexibility to select technologies that best meet their site-specific needs, including regulatory requirements, budget constraints, and environmental conditions. However, the prevalence of multiple brands and systems can sometimes create confusion, especially for small communities with limited technical expertise.^52^ Understanding these core principles helps small communities navigate the marketplace and choose systems that align with their specific goals, beyond marketing claims.

#### 2.3.4 Installation Location

Technologies were also categorized by installation location: prelagoon, in-lagoon, or add-on/postlagoon. Prelagoon systems, placed upstream, reduce nutrient loads entering the lagoon, protecting treatment efficiency. Though effective, they require added infrastructure, like reactors or chemical dosing units, which increases costs and complexity, posing challenges for small communities.^9^ In-lagoon technologies enhance removal directly within the lagoon using aeration, bioaugmentation, or chemical dosing. These often leverage existing infrastructure, offering cost-effective upgrades.^45^ However, energy demands and environmental sensitivity, particularly in cold climates, can affect reliability.^53^

Add-on/postlagoon technologies, installed downstream, provide advanced polishing to meet discharge limits. Examples include wetlands, filters, algae systems, and chemical units.^49^ They offer high removal efficiency but require more land, energy, and skilled maintenance, factors that strain limited budgets.^5^ A variant is side-stream treatment, where a portion of lagoon effluent is diverted for targeted nutrient removal, minimizing disruption. Technologies like struvite precipitation or anammox reactors offer modular options for phosphorus and nitrogen recovery, respectively.^48,54^ While side-streams demand infrastructure and monitoring, they can be more affordable than full upgrades and offer a phased path to compliance.^38^

#### 2.3.5 Development Stage

Technologies were classified by their development phase, bench-scale, pilot-scale, or full-scale. This helps identify not only technical maturity but also economic readiness. The availability of nutrient management technologies on the market heavily depends on their development phase. Bench-scale technologies often remain experimental and are rarely commercially available. Pilot-scale technologies may be offered on a trial basis by developers seeking additional performance data, while full-scale technologies are generally market-ready but come with higher costs due to the need for extensive infrastructure and technical support.^30^ While the reliability of performance data improves significantly as a technology advance through these phases, so do the associated costs. This makes careful evaluation of scalability and economic feasibility critical before committing to widespread adoption of these technologies in lagoon systems.^55^

In summary, this categorization framework enabled a comprehensive and comparative evaluation of nutrient management strategies for lagoon systems. By aligning each technology with its core process, nutrient focus, application setting, and maturity level, this review offers decision-makers actionable insights to support effective, sustainable, and context-specific nutrient management in small-community lagoon systems.

### 2.4 Suitability Index

Selecting the most appropriate nutrient management technology for lagoon systems in small communities often revolves around two primary considerations: performance and cost. While these factors are critical, they do not fully capture the broader range of elements influencing the feasibility and sustainability of implementing technologies, particularly in small communities with unique challenges. To address this, the Suitability Index (SIDX) was developed as a holistic evaluation framework, integrating both technical and contextual criteria into a single metric for decision-making.

The SIDX assessed technologies based on nine key factors: existing infrastructure, process complexity, automation, market availability, site factors, consumables, operational dependability, maintenance, and target nutrient removal. Temperature dependence was included under the ‘site factors’ criterion which required that technologies demonstrate stable performance even under low-temperature conditions. While most lagoons in the U.S. are located in temperate regions, for those situated in colder areas seasonal variation can significantly impact performance. These nine factors were chosen to assess the viability of nutrient management technologies in small community lagoon systems, aligning with essential criteria for sustainable water treatment facilities.^56^

Each technology was assigned relative scores based on its performance across these criteria. Notably, all three nutrients (ammonia, TN, and TP) were weighted equally, with scores assigned according to the number of target nutrients that a technology could address. To ensure consistency and reliability, the scores were weighted using the Analytic Hierarchy Process (AHP), which involved constructing a pairwise comparison matrix, calculating relative weights through matrix normalization, and performing consistency checks. The verification process included calculating the consistency ratio to confirm that it remained below the acceptable threshold of 0.10, ensuring robust weight assignments.^57,58^ The final SIDX aggregates were normalized on a 0–1 scale. For better interpretability, an indexed scale (0–100) was provided, where the maximum observed SIDX corresponds to 100. This was used to rank technologies, offering a simplified framework to help small communities quickly identify the most suitable options from a wide range of available solutions. A detailed methodology on the development of the AHP pairwise matrix and subsequent SIDX is provided in the Tables S4–S6.

## 3 Results and Discussion

The literature review reveals a clear evolution in research priorities for nutrient removal technologies over the past five decades (Figure 3). In the 1960s and 70s, research was dominated by bench-scale studies focused on evaluating feasibility, optimizing treatment conditions, and exploring new nutrient removal concepts under controlled settings.^59^ Over subsequent decades, pilot-scale studies steadily increased, bridging the gap between lab-based innovation and field application by addressing real-world operational challenges and refining system designs. Since the late 2000s, full-scale studies have grown significantly, reflecting a shift toward the practical deployment of nutrient removal technologies.^47,60^ This trend signals both the maturation of the field and the translation of successful earlier-phase research into implementable solutions. It may also be attributed to increasingly stringent nutrient discharge limits over time which have heightened demand for scalable, high-performance systems.^18^

**Figure 3:**
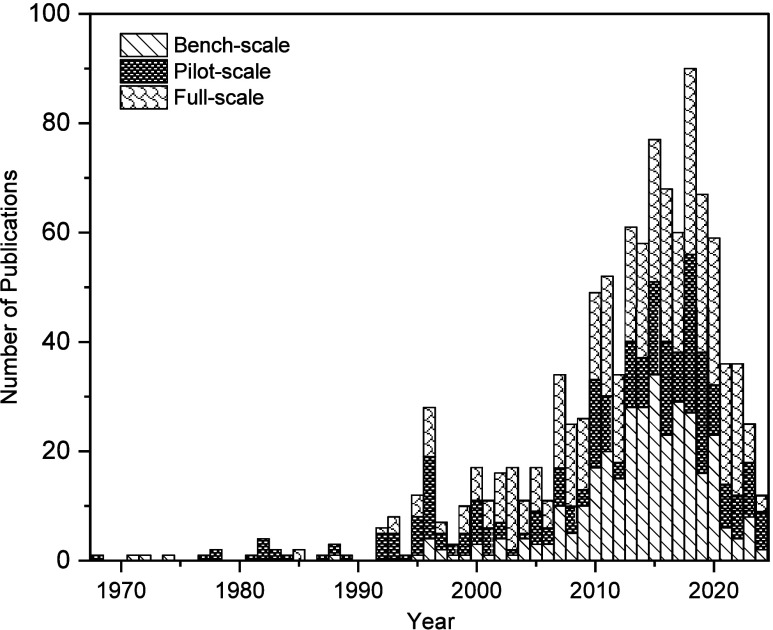
Development of nutrient management strategies applicable to lagoons in small communities over the last five decades.

Among the 1216 nutrient management strategies identified, biological processes emerged as the most prevalent, comprising 79% of applications (Figure 4). This dominance reflects their effectiveness, cost-efficiency, and alignment with the operational realities of small communities. Their ability to target multiple nutrients simultaneously and integrate into existing configurations makes them particularly suitable for small community lagoon systems.^28,52^

**Figure 4:**
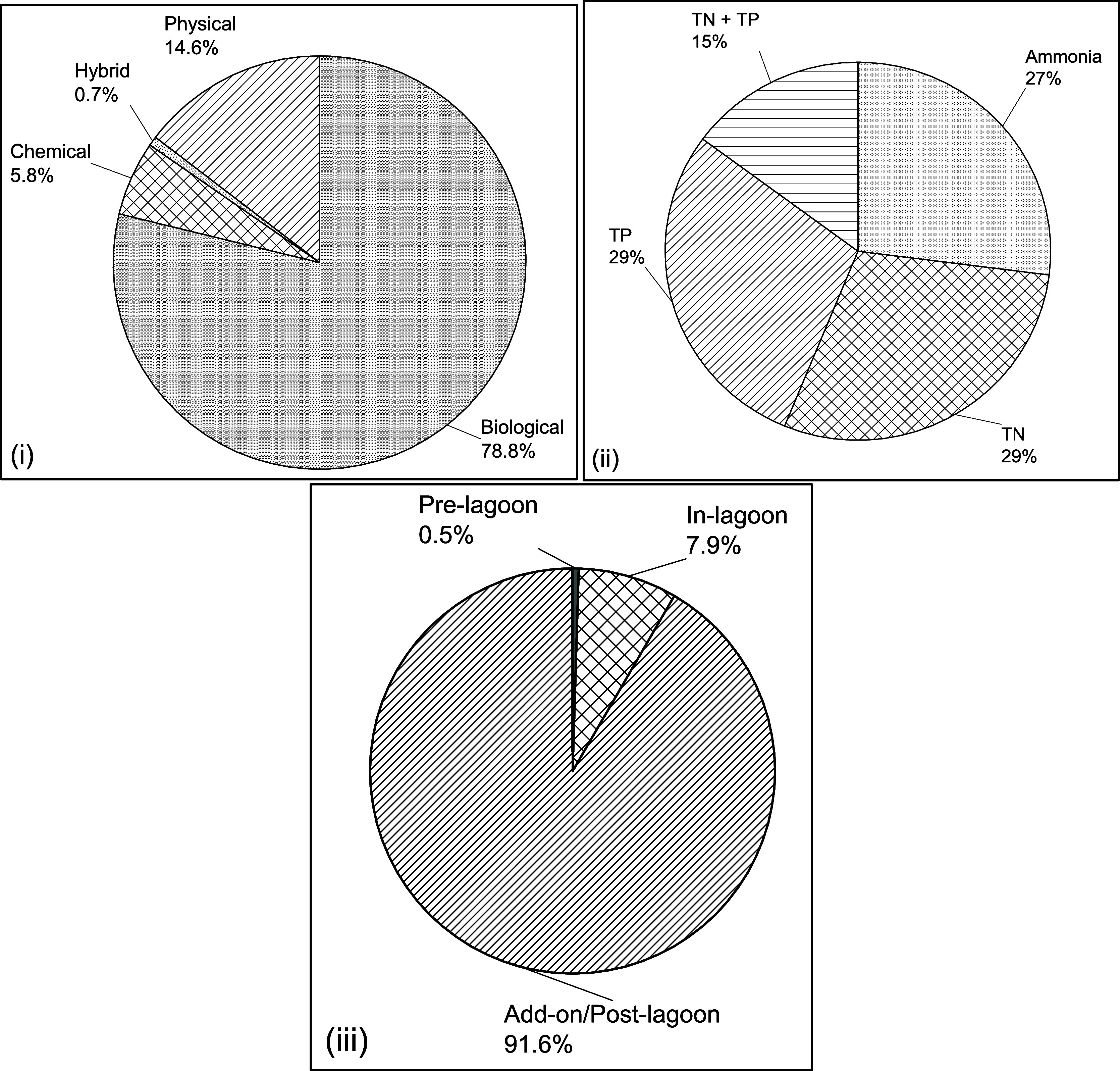
Distribution of nutrient management strategies based on (i) process classification, (ii) type of nutrient removed, and (iii) installation location. *N* = 1216.

Chemical processes make up a smaller share (5%), primarily employed for targeted applications such as phosphorus removal or as polishing steps to meet stringent effluent standards. Despite their effectiveness, the reliance on consumables and higher operational costs limits their broader adoption.^45^ Physical processes and hybrid methods account for 15 and 1%, respectively, indicating their supplementary roles, such as pretreatment or enhancing biological and chemical operations.^61,62^ This distribution highlights the centrality of biological processes in nutrient removal strategies, while chemical and physical methods play vital, albeit more specialized, roles in addressing specific challenges or providing additional treatment capabilities.

Technologies addressing combined TN and TP removal accounted for approximately 28%, while those targeting ammonia-only, TN-only, and TP-only removal constituted 30, 23, and 19%, respectively. The dominance of ammonia-focused technologies can likely be attributed to the historical regulatory emphasis on controlling ammonia levels in wastewater, which may have driven extensive research and development in this area. Conversely, the relatively lower prevalence of TP-focused technologies may reflect limited regulatory pressure on phosphorus management during the same period, resulting in fewer instances documented in the literature.^18,63–65^ Regarding installation location, the technologies were overwhelmingly classified as “add-on” systems (92%), with smaller contributions from “in-lagoon” (7%) and “pre-lagoon” (1%). This trend suggests that most nutrient removal technologies are designed as supplementary systems to existing infrastructure, enhancing operational flexibility albeit with potential costs associated with retrofitting or modifying lagoon systems.^45,59^

### 3.1 Technology Classifications

A key challenge encountered during the review was the sheer number and diversity of nutrient management technologies, often described using inconsistent or overlapping terminology. This lack of standardized classification created difficulties in comparing systems across studies and posed a barrier to interpretation, particularly for small communities with limited technical capacity seeking practical, actionable guidance. To address this issue, the review refined existing process classifications by organizing the 1216 identified instances of nutrient management strategies into 23 distinct underlying technologies thereby minimizing variability introduced by commercial branding and consolidating approaches with shared treatment mechanisms. These were further grouped into four overarching categories based on treatment approach and operational complexity within the context of lagoon wastewater treatment: (1) conventional systems, (2) enhanced biological systems, and (3) advanced technologies (Figure 5).

**Figure 5:**
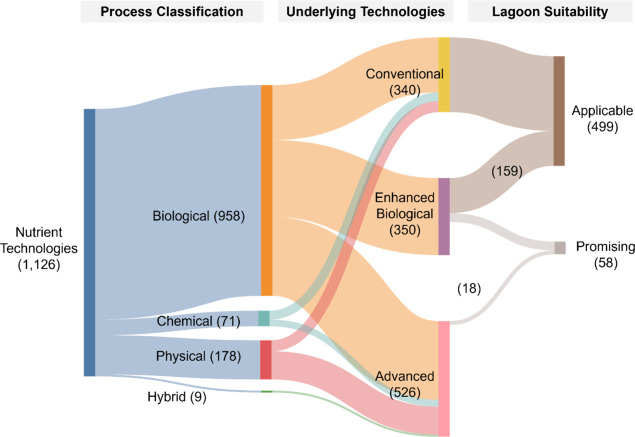
Classification of nutrient management technologies identified in the literature review and their suitability in small community municipal lagoons. Band thickness and numbers in parentheses represent the number of technologies in each category. The rightmost side indicates whether technologies have been demonstrated or discussed for use in lagoon contexts. Conventional and enhanced biological technologies are generally applicable, with some enhanced biological technologies considered “promising.” Although most advanced technologies are not yet viable for small community lagoon applications, several still hold potential. For additional detail on representative process schematics of key technologies discussed, see EPA (2011) and Qasim and Zhu (2017) for aerated and attached-growth systems; EPA (2000) and Kadlec and Wallace (2009) for wetland systems; and Ødegaard (2006) for moving bed biofilm reactors.

#### 3.1.1 Conventional Technologies

Conventional technologies refer to proven, widely adopted methods with established implementation in lagoon systems. These include aeration, chemical precipitation, maturation ponds and constructed wetlands. Aeration systems in lagoons achieved average ammonia removal efficiencies of 76 ± 15% and TN removal efficiencies of 53 ± 13% (Figure 6). Aeration enhances microbial activity, promoting nitrification, the biological conversion of ammonia to nitrate, which improves ammonia removal.^1,60,63^ However, complete TN removal also requires complementary denitrification processes that are often absent in typical lagoon configurations.^44^ Oxygen transfer efficiency varies across aeration methods: diffused aeration offers better distribution but involves higher complexity and cost, while surface aerators are simpler and more economical to operate.^66,67^ Variability in nitrogen removal performance largely stems from different aeration methods and operations.^68^ Nonetheless, aeration remains a practical, low-cost upgrade for facultative lagoons, improving both ammonia and BOD removal and making it especially valuable for small communities aiming to enhance treatment performance within existing infrastructure.^59,69^

**Figure 6:**
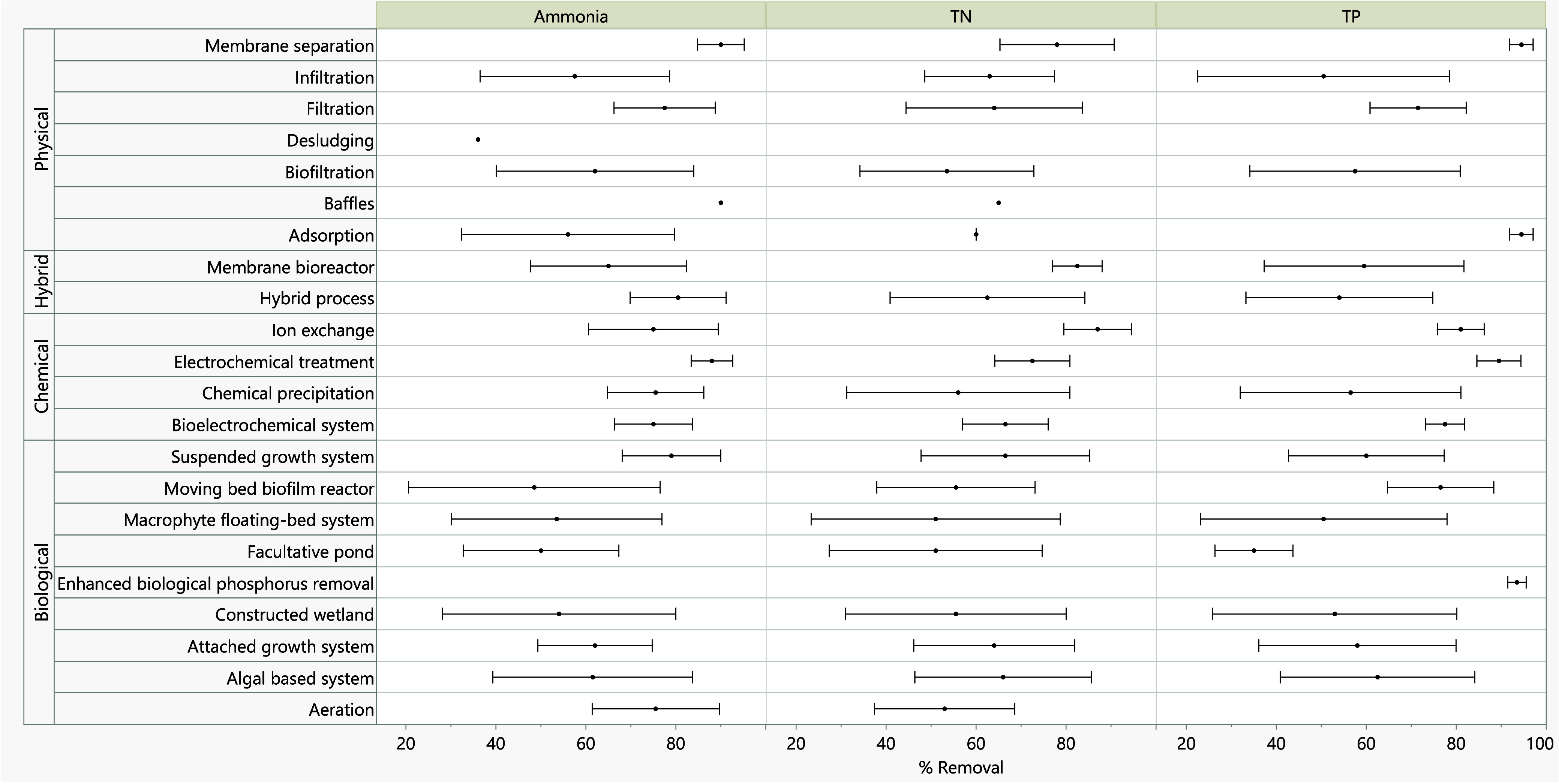
Removal efficiencies of underlying technologies for ammonia, total nitrogen, and total phosphorus removal.

Chemical precipitation emerged as a widely utilized technology, achieving average removal efficiencies of 57 ± 27% for TP, 76 ± 11% for ammonia, and 56 ± 20% for TN. The process involves the addition of chemical agents such as alum, ferric chloride, or lime, which cause phosphorus to precipitate into insoluble forms that are subsequently removed via sedimentation.^48,70^ In addition to phosphorus removal, the increase in pH during dosing can indirectly promote ammonia volatilization, contributing to improved ammonia removal.^71^ Chemical precipitation systems are often implemented as modular add-ons to existing lagoon infrastructure, providing targeted phosphorus control with minimal structural modification.^1^ However, these systems generate sludge requiring proper disposal and incur recurring costs for reagents, posing challenges for resource-limited communities.^38^ Observed performance variability may be attributed to several factors including pH control, chemical dosing precision, and mixing efficiency.^45,47^

Maturation ponds, accounting for 8% of identified technologies (100 instances), commonly serve as cost-effective polishing units for lagoons. These systems enhance effluent quality by promoting UV disinfection and nutrient removal through sedimentation and biomass assimilation.^20^ With 66% implemented at full scale, maturation ponds are well-established, low-maintenance options particularly suited for small communities. They can be easily integrated downstream of existing lagoons, requiring minimal operational expertise.^9,72^ However, their reliance on natural processes can result in variability in performance, especially under cold climate conditions where treatment efficiency may decline.^72^

Constructed wetlands (CWs) emerged as a prominent solution, representing 12% of total technologies. CWs achieve nutrient removal through plant uptake, filtration, and biomass assimilation, with ammonia removal efficiencies of 54 ± 25%, TN removal of 56 ± 20%, and TP removal of 53 ± 30% (Figure 6). This wide variability in nutrient removal efficiencies is influenced by design, seasonal climate variations, which affect microbial activity and plant uptake, as well as maintenance practices, where issues like clogged media or poor hydraulic flow can further impact ammonia, TN, and TP removal.^73^ For instance, vertical subsurface flow wetlands are particularly effective due to improved aeration, while horizontal flow wetlands are robust and suitable for larger hydraulic loads.^74^ Hybrid CWs, combining vertical and horizontal flow systems or other technologies such as microbial fuel cells or adsorbents, enhance nutrient removal up to 90% by optimizing aerobic and anaerobic processes.^14,75^ CWs can integrate seamlessly with lagoon systems, offering additional nutrient removal capacity and cobenefits such as habitat creation and stormwater retention.^76^ For small communities, CWs can be tailored to limited land availability by prioritizing vertical or hybrid designs and integrating them into existing lagoon layouts. However, they may require routine maintenance to prevent clogging and remain sensitive to climate-driven shifts in plant growth and microbial activity.^77^

#### 3.1.2 Enhanced Biological Systems

Enhanced biological systems refer to those that combine natural processes with engineered components to improve nutrient removal. This category includes suspended growth systems, attached growth systems, macrophyte floating bed (frequently referred to as floating wetlands) and algal systems. Suspended growth systems were prominent in the review, with approximately 180 documented applications, including sequencing batch reactors (SBRs) and complete-mix aeration ponds.^28,61^ These technologies rely on suspended microbial activity and biomass assimilation, achieving ammonia removal efficiencies of 70–90% and TN removal of 60–80%.^23,61^ Their adaptability makes them suitable as treatment upgrades for existing lagoons in small communities aiming to meet stricter effluent standards. Compact configurations such as SBRs are especially advantageous in land-constrained settings due to their high treatment capacity and minimal spatial footprint.^44^ However, their high energy demands for aeration and mixing, coupled with complex operation and maintenance requirements, pose financial and technical challenges for small, resource-limited communities.^54,70,78^

Attached growth systems, including rotating biological contactors (RBCs), trickling filters, and moving bed biofilm reactors (MBBRs), represented 7% of identified technologies. These systems enhance nutrient removal by promoting biofilm development on fixed or mobile media, achieving average ammonia, TN, and TP removal efficiencies of 62 ± 10, 64 ± 15, and 58 ± 23%, respectively.^79,80^ MBBRs, which integrate suspended and attached growth mechanisms, offer higher treatment efficiency by increasing microbial surface area. Their modularity and scalability allow easy integration into existing lagoon systems as polishing or secondary units, particularly for small communities seeking cost-effective upgrades. However, their performance is influenced by media type, flow patterns, and operational conditions, and regular maintenance is required to prevent clogging and ensure optimal biofilm activity.^81,82^ Despite these considerations, attached growth systems remain practical, adaptable solutions for enhancing lagoon nutrient management.

Macrophyte floating bed systems, widely documented in the literature, remove nutrients through plant uptake, microbial activity in root zones, and natural filtration.^83,84^ These systems show strong potential for phosphorus removal, often exceeding 60% under optimal conditions, and are particularly suited for small communities with large lagoon surface areas due to their minimal infrastructure needs and passive operation. Additional benefits include habitat creation and aesthetic enhancement.^85,86^ However, their effectiveness is limited in colder climates or during low sunlight periods, and performance can be variable, with pilot studies showing TP removal between 40 and 70%.^87^ Regular maintenance, such as plant harvesting and managing root zone buildup, is necessary to sustain function. While cost-effective and ecologically beneficial, their dependence on favorable climate and space makes them less suitable for constrained or colder regions.^88,89^

Algal-based systems, representing 16% of the technologies identified, utilize algal-bacterial assimilation to remove nutrients, with reported efficiencies of 62 ± 20% for ammonia, 66 ± 19% for TN, and 63 ± 21% for TP. Unlike conventional facultative lagoons, which passively support algal activity, these systems are purposefully engineered to enhance algal growth and nutrient uptake.^90^ Well-suited for lagoon integration, particularly in sun-rich regions, they harness natural processes for efficient nutrient removal.^91,92^ Common configurations include suspended, immobilized, and attached algae systems, offering flexibility in design and operation.^93^ Suspended algal systems, such as high-rate algal ponds, allow algae to grow freely within the water column, supporting high productivity. However, they require efficient harvesting methods and careful hydraulic management to prevent washout.^22,94^ Immobilized systems, where algae are encapsulated or embedded in a supporting matrix, provide better control over nutrient uptake, reduce washout risks, and simplify harvesting, but they involve higher material and operational costs.^95^ Attached algal systems, such as algal turf scrubbers, rely on algae growing on fixed surfaces, offering operational simplicity and resilience to hydraulic disturbances but requiring careful design to ensure adequate light and nutrient availability.^92,96^ Performance across all systems is sensitive to light, temperature, and nutrient availability, with immobilized and attached designs offering greater stability but requiring optimized conditions and routine maintenance.^71,97^

#### 3.1.3 Advanced Technologies

Advanced systems offer high-performance nutrient removal through specialized designs, though they often require greater operational oversight and technical expertise. This category includes anaerobic pretreatment systems and electrochemical treatment technologies, both of which are less common in small-community applications but show strong potential under the right conditions. Anaerobic pretreatment systems, such as anaerobic baffled reactors (ABRs) and upflow anaerobic sludge blanket (UASB) reactors, and anammox systems leverage anaerobic digestion and ammonia oxidation to reduce nutrient loads. ABRs achieve high nitrogen removal efficiencies of 89 ± 3% by maintaining extended contact between wastewater and anaerobic biomass, while UASB reactors promote the formation of granular sludge in compact reactor configurations.^98,99^ In addition to biological conversion, anaerobic systems support nutrient reduction through sludge wasting. Despite their efficiency, these systems face significant implementation barriers in lagoons, including long microbial start-up times, temperature sensitivity, and the need for skilled monitoring and operational control.^100,101^ Other cobenefits of anaerobic systems include reduced sludge production, biogas recovery and improved organic load management, making them a potential, though technically demanding, option for future application in lagoon-based treatment.^102,103^

Bioelectrochemical systems, including microbial fuel cells (MFCs) and microbial electrolysis cells (MECs), are emerging technologies that integrate microbial metabolism with electrochemical processes to achieve nutrient removal alongside energy recovery.^104,105^ These systems have demonstrated ammonia removal efficiencies of 75 ± 11%, TN removal of 67 ± 14%, and TP removal of 80 ± 5%. Their modular nature allows for integration into existing lagoon systems, particularly in anaerobic or aerobic zones, making them applicable for small community applications. However, high capital costs, operational complexity, and the need for skilled oversight currently limit their deployment beyond the pilot scale.^106,107^

Similarly, electrochemical treatment systems, including electrolysis and electrodialysis, demonstrate high nutrient removal efficiencies, 88 ± 8% for ammonia, 73 ± 10% for TN, and 90 ± 5% for TP, making them particularly effective for selective ion removal and effluent polishing.^108^ These modular systems can be integrated into lagoon systems as standalone polishing steps to improve effluent quality. However, energy demands, membrane fouling, and the need for optimized water chemistry present operational challenges.^109^ Like bioelectrochemical systems, most electrochemical treatment systems are at the pilot scale, with limited commercially available options suitable for small communities.^110^ Nevertheless, the high performance of these technologies positions them as valuable upgrades for enhancing lagoon efficiency as they advance toward full-scale, cost-effective deployment.

Ammonia stripping, anammox, and enhanced biological phosphorus removal (EBPR) also featured in the review, though primarily at bench or pilot scale. These technologies are typically designed for large mechanical systems, and while they show strong potential under controlled conditions, their application in lagoon settings remains largely untested and operationally challenging. Anammox systems have demonstrated ammonia removal of 90 ± 5% and TN removal of 87 ± 2%, under tightly controlled anaerobic conditions, but their application is limited by long microbial startup times and temperature sensitivity.^101,111^ Ammonia stripping, reported in 15 studies, achieved 89 ± 9% ammonia and 72 ± 27% TN removal, typically via pH adjustment (above 10) and heating to volatilize ammonia.^20,49^ Although conceptually adaptable to lagoons as modular side-stream units or post-treatment steps, the need for precise pH control, energy input, and gas handling infrastructure poses significant feasibility challenges in low-tech lagoon systems.^44,112^ EBPR systems which enrich polyphosphate-accumulating organisms under alternating aerobic and anaerobic conditions, achieved TP removal of 94 ± 4% in 12 studies.^113^ While theoretically adaptable to lagoons through cell modifications or compact postlagoon units, they require consistent influent quality, volatile fatty acid availability, and skilled oversight, making practical implementation in lagoons difficult under current conditions.^47^

### 3.2 Hydraulic Improvements

Hydraulic adjustments in lagoon systems, such as baffle installation and regular desludging to maintain lagoon profiles, featured as strategies for optimizing performance and supporting nutrient management. Baffle systems, while not intended as primary nutrient removal technologies, significantly enhance TN removal efficiency (up to 90%) and TP removal (up to 65%) by improving flow distribution and increasing contact between wastewater and treatment processes, including microbial activity and algal growth.^1,23^ By reducing short-circuiting and increasing hydraulic retention time, baffles improve overall system efficiency and ensure consistent treatment performance.^114,115^ Similarly, desludging, though not directly targeting nutrients, removes sediment-bound nutrients, achieving TN removal efficiencies of around 36%, and helps maintain lagoon depth, prevent resuspension of solids, and sustain system stability.^116^ Together, these simple yet impactful interventions enhance the hydraulic efficiency and long-term resilience of small community lagoon wastewater treatment systems.

### 3.3 Circularity and Resource Recovery

While nutrient removal has long been the dominant focus in lagoon-based systems, recent research increasingly reflects a paradigm shift toward circularity, resource recovery, and climate-aligned wastewater management. Although this review identifies many technologies traditionally deployed for nutrient removal, a substantial subset of studies explored strategies that transform wastewater nutrients into recoverable resources, offering cobenefits such as reduced reliance on synthetic fertilizers, energy recovery, and emissions offsets. These advances are particularly relevant as the water sector moves toward net-zero carbon targets and integrates circular economy principles into utility operations.

Land application of treated effluent, one of the oldest and most studied nutrient recovery strategies (169 studies), offers a low-energy method to recycle nitrogen and phosphorus for agricultural use while conserving freshwater and enhancing soil fertility.^1,42^ Despite its benefits, adoption remains limited due to seasonal constraints, land availability, regulatory hurdles, and concerns about pathogens and residual contaminants.^117,118^ Where feasible, it remains a practical and sustainable option for rural communities seeking cost-effective nutrient reuse.

Struvite precipitation featured in the study as a nutrient management technology for phosphorus and ammonium recovery, with multiple studies demonstrating its feasibility as a post-treatment step in decentralized systems.^17,119^ When optimized through chemical dosing and pH adjustment, the process yields magnesium ammonium phosphate (struvite), a slow-release fertilizer that is more sustainable than conventional chemical alternatives.^120^ Although traditionally applied to high-strength wastewater, newer low-footprint reactor designs have improved viability in small-scale and decentralized contexts.^47^ However, its specialization in treating concentrated waste streams makes struvite precipitation largely unsuitable for lagoon systems, where influent nutrient concentrations are typically too diluted to enable efficient recovery. Nonetheless, struvite precipitation contributes to climate goals by reducing reliance on energy-intensive, synthetic fertilizer production.^121^

Algal-based systems (144 studies) exemplify circularity by coupling nutrient uptake with biomass production, which can be converted into bioenergy, soil amendments, or animal feed.^96,122^ Technologies like rotating algal systems, high rate algal ponds and attached turf scrubbers are particularly suited for small communities with ample sunlight and land availability.^92,95^ While often regarded as environmentally friendly, these systems can emit methane and nitrous oxide, especially during biomass decomposition or periods of high productivity, posing a significant trade-off.^123^ To align with net-zero goals, mitigation strategies such as frequent harvesting or integrating anaerobic digestion may be needed.^124^

Wastewater aquaculture (54 studies) represents another biologically integrated strategy that links nutrient treatment with biomass reuse. Systems such as solar aquatic ecosystems, aquaculture wetlands, and integrated multitrophic aquaculture (IMTA) use treated effluent to cultivate aquatic plants or fish, generating outputs like duckweed, which can be used as livestock feed or soil conditioner.^125,126^ These systems exemplify circular economy principles by transforming waste into resources. However, their broader adoption is limited by health concerns, regulatory hurdles, and market acceptance, particularly in informal or small-scale settings.^127^

Additionally, anaerobic reactors (26 studies) offer an integrated approach to organic and nutrient reduction while producing methane-rich biogas that can be recovered for local energy use. Particularly attractive for off-grid communities, these systems can reduce sludge production and improve downstream lagoon performance by lowering organic loading.^100,128^ Their use as a pretreatment unit supports both operational efficiency and climate mitigation goals by offsetting fossil energy and supporting codigestion of high-strength organic waste streams.

It is important to note that, beyond nutrient removal performance, nutrient management in lagoons also has greenhouse gas emission implications. Anaerobic processes can be notable sources of methane, while nitrification–denitrification processes may release nitrous oxide, with stringent dissolved oxygen control often impractical in small community contexts.^41,121^ In addition, indirect emissions from construction materials, energy use, and transportation contribute further to the overall carbon footprint.^129^ However, only a limited number of studies have directly investigated GHG emissions from lagoons, and the estimation methods applied vary, including direct measurement of CH_4_ flux, mass-balance approaches, process-based modeling, and the use of simple emission factors, with the latter being most common.^130–132^ Reported values typically range from 0.4 to 3.1 kg CO_2_-eq per m^3^ of treated wastewater, although substantial uncertainty remains.^130,133,134^ Collectively, these direct and indirect emissions underscore that nutrient management decisions in lagoons must be evaluated not only in terms of nutrient removal effectiveness and cost, but also with respect to their environmental impacts. Future research is needed to more accurately quantify the life cycle carbon footprint of lagoons and generate robust data to support decision-making in small community contexts.

In summary, the progression of nutrient management technologies reflects a range of established methods together with advanced and specialized innovations. However, the high standard deviations reported for nutrient removal efficiencies highlight substantial variability across treatment technologies. This variation stems from differences in system design (e.g., suspended vs attached growth), media characteristics, scale (pilot vs full-scale), and flow regime (batch vs continuous) Biological processes, such as microbial and plant-based nutrient uptake, are also highly sensitive to temperature, pH, and light, which vary seasonally and geographically.^1,42,53,135^ Operational capacity further influences performance, with skilled staffing, routine maintenance, and monitoring all affecting treatment reliability. Inadequate oversight can lead to chemical underdosing or biomass buildup, reducing system effectiveness.^45^ These challenges are often more acute in resource-limited settings with constrained technical support, as is typical in small communities.^52^

### 3.4 Suitability for Small Communities

Given the wide range of nutrient management strategies identified, spanning different stages of development and performance, it was necessary to assess their applicability to small communities that rely on municipal lagoon systems. As previously noted, average nutrient removal values can be misleading due to the high variability in performance across technologies. Therefore, decision-makers must consider not only nutrient removal efficiencies but also factors such as availability, environmental sensitivity, and operational requirements to determine local suitability. To support this need, a Suitability Index (SIDX) was developed, combining factors such as technical maturity, implementation feasibility, and environmental compatibility into a single score ranging from 1 (least suitable) to 100 (most suitable).

A comparison of SIDX scores across development stages shows a clear trend: technology suitability improves as systems advance from bench to pilot and full-scale deployment (Figure S4). Mean SIDX values increased from 50 at bench scale to 61 at full scale, with the maximum reaching 100, reflecting greater feasibility and validation at later stages.^28,30,55^ However, variability remains high, influenced by classification breadth and context-specific performance. For example, suspended growth systems span diverse configurations leading to wider SIDX ranges compared to more standardized processes like chemical precipitation, which are more uniform in design and operation.^70^ Based on alignment with lagoon operations, technologies were grouped into three categories: applicable, promising, and not currently viable (Figure 7).

**Figure 7:**
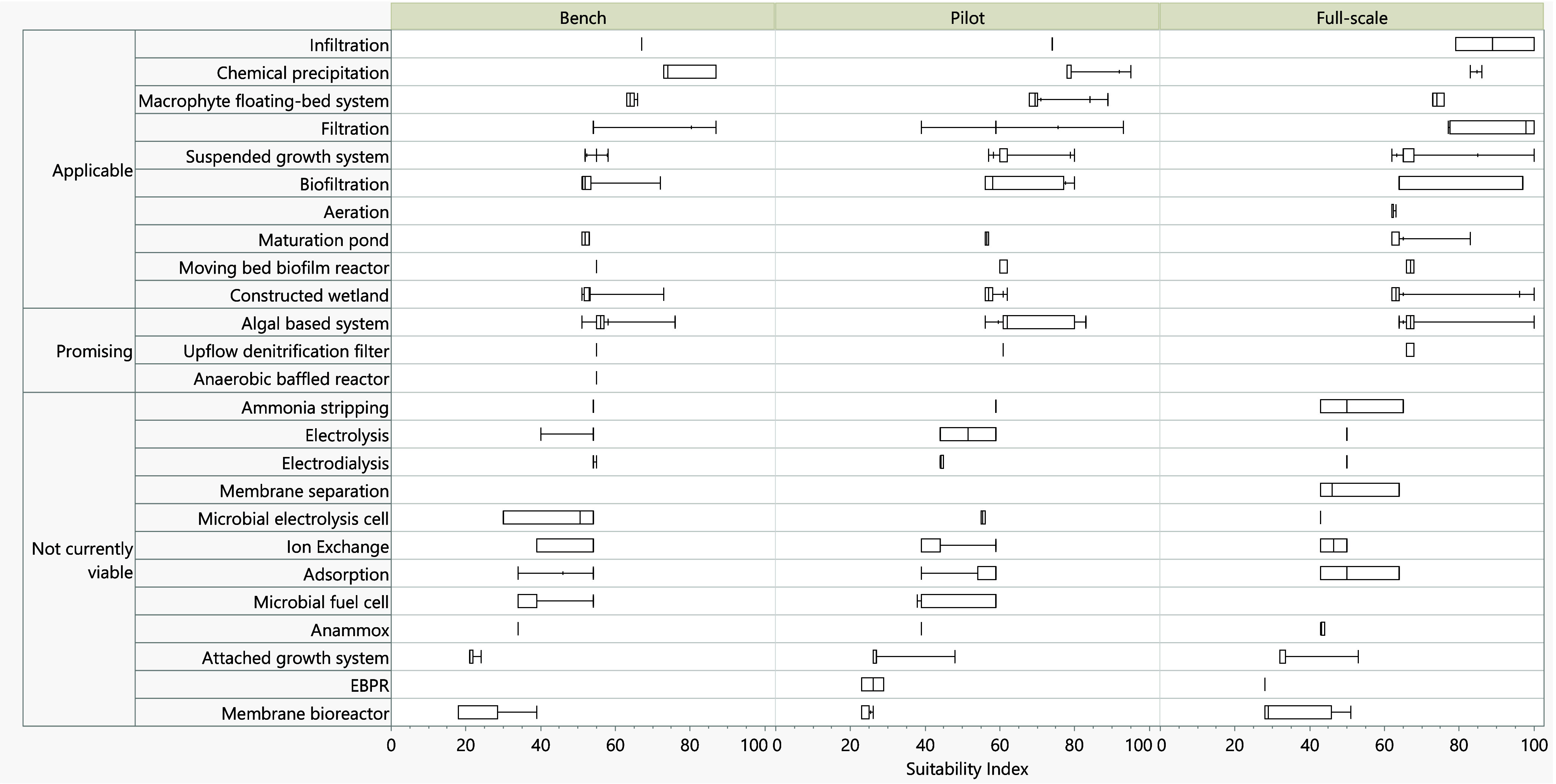
Box plots of suitability indices (1–100) of underlying nutrient management technologies across different phases of development.

#### 3.4.1 Applicable Technologies

Applicable technologies demonstrate robust field performance and full-scale deployment success, with normalized weighted score aggregate >0.15 (corresponding to SIDX > 62 in the (1–100 scale). This threshold was selected because it corresponds to the inflection point of the SIDX score distribution and identifies options that either address two or more nutrients or pair one-nutrient coverage with strong feasibility attributes (lagoon-ready, market availability, and minimal O&M). These systems align well with the low-energy, passive operations common in small community lagoon systems. Partial-mix aeration systems (SIDX ∼ 60), though underrepresented in the literature, are widely used to enhance nitrification and BOD removal by increasing microbial activity and oxygen availability.^59^ They offer a practical, low-cost upgrade to existing lagoons, improving ammonia removal without significantly altering infrastructure.^136^ Similarly, suspended growth systems such as sequencing batch reactors and complete-mix lagoons (SIDX 60–64) demonstrate strong performance in nitrogen removal, though advanced configurations may require increased operational oversight and process control.^137^

Constructed wetlands (SIDX up to 84) are among the most adopted nature-based systems, representing 12% of total technologies. The extensive use of wetlands is attributed to ease of operation and low energy requirements.^75^ Future evaluations may benefit from distinguishing between subsurface, free water surface designs and other types of wetlands, given their different climate tolerances and flow dynamics. Macrophyte floating-bed systems (SIDX 71–74), meanwhile, perform well in warmer regions but face reliability concerns under seasonal fluctuations and require regular maintenance.^89^ Maturation ponds (SIDX ∼ 62) serve as simple polishing steps that enhance effluent quality, though they may underperform in colder climates.^49^

Additional applicable solutions include biofiltration and packed-bed filters (SIDX 61–63), which aid in nitrification and algae control, especially under low-flow, postlagoon conditions.^31^ Chemical precipitation (SIDX ∼ 82) is highly effective for phosphorus removal using alum or ferric salts, though its success depends on precise dosing and sludge handling.^47^ Moving Bed Biofilm Reactors (MBBRs) (SIDX ∼ 66) combine suspended and attached growth modes, offering modular integration into existing lagoon infrastructure.^82^

Infiltration and land application methods (SIDX ∼ 98) support nutrient reuse while reducing discharge volumes.^118^ Desludging (SIDX ∼ 76), though not a direct nutrient removal technique, minimizes internal nutrient cycling by removing settled solids.^116^ Lastly, baffles (SIDX ∼ 86) improve hydraulic retention and flow distribution, though their SIDX score may reflect ease of implementation more than nutrient removal efficiency.^115^

#### 3.4.2 Promising Technologies

Technologies categorized as promising, scored less than 59 on the Suitability Index (SIDX) and exhibit potential for future integration into lagoon systems under favorable conditions. Among them, algal-based systems (SIDX ∼ 55), including suspended, immobilized, and attached configurations, facilitate nutrient removal through photosynthesis and algal-bacterial assimilation. While their performance is inherently sensitive to light availability and temperature, emerging designs that incorporate greenhouse enclosures offer enhanced climate resilience and more consistent year-round operation, improving their reliability and viability for small communities with suitable environmental conditions.^92,96^

Upflow and horizontal flow denitrification filters (SIDX ∼ 35) offer a viable option for postlagoon nitrate polishing, particularly when paired with external carbon dosing or solid-phase carbon sources such as wood chip bioreactors. Wood chip bioreactors provide a low-cost, passive means of supplying carbon to denitrifying microorganisms. Their potential advantages in lagoon applications include long media life, low operation and maintenance demands, and the ability to achieve sustained nitrate removal without continuous chemical addition. However, challenges remain around achieving full nitrification in cold conditions, hydraulic control, the gradual depletion of carbon over time, and potential leaching of organics during startup. Adapting wood chip filters for lagoon effluents may therefore require careful design of residence times and periodic media replacement.^31,81^ Anaerobic baffled reactors (SIDX ∼ 32) and upflow anaerobic sludge blanket (UASB) systems (SIDX ∼ 43) contribute to nutrient management primarily through solids reduction and sludge wasting, rather than direct removal of soluble nutrients.^128^ These systems can effectively lower organic loads and support biogas recovery, aligning with circular treatment goals. However, their broader application in small community lagoons is constrained by temperature sensitivity, extended microbial startup times, and the need for consistent operator oversight and training, challenges that must be addressed before widespread implementation becomes feasible.^98^

#### 3.4.3 Technologies Not Currently Viable

Technologies were considered unsuitable for widespread use in small lagoon-based systems due to their high complexity, energy demands, or incompatibility with dilute influents. Anammox (SIDX 43), while effective under controlled conditions, is poorly suited for lagoons due to its reliance on strict anaerobic environments and slow microbial growth.^111^ Similarly, ammonia stripping (SIDX 39–56) is operationally intensive, requiring elevated pH, heating, and air stripping, challenges that are difficult to manage in lagoon systems.^107^ Both technologies are most appropriate for high-strength wastewater and face significant barriers to adoption in lagoon contexts.

Enhanced Biological Phosphorus Removal (EBPR) (SIDX 28) is also constrained by the need for alternating aerobic and anaerobic zones, which are not typical of passive lagoon operations.^51^ Ion exchange, adsorption, and struvite precipitation (SIDX 39–46) were evaluated as too costly and ineffective for the dilute nutrient loads present in lagoon effluent.^50,119^ These methods are better suited to high-strength wastewater streams and require tight operational control, which may be beyond the reach of small utilities.

Bioelectrochemical approaches, including microbial fuel cells (MFCs) and microbial electrolysis cells (MECs) (SIDX 30–39), offer the dual benefit of nutrient removal and energy recovery but remain largely at the pilot scale. These systems depend on stable influent quality and high concentration gradients, conditions rarely met in lagoon effluent, limiting their practical applicability.^104^ While bioelectrochemical systems show theoretical promise in research settings, but without significant improvements in reliability, automation, and cost, they remain unsuitable for field deployment in decentralized or resource-limited contexts.^106^ More advanced electrochemical processes, including electrolysis, electrodialysis, and other general electrochemical treatments (SIDX 46–57), share similar constraints; despite strong removal potential, their operational complexity and power requirements exceed the capacity of typical lagoon systems.^110^

Membrane bioreactors (MBRs) and membrane separation technologies (SIDX 24–33) also demonstrate high nutrient removal efficiency but are hindered by challenges such as membrane fouling, frequent maintenance, and high energy demands, making them cost-prohibitive for small communities.^61^ Attached growth systems such as trickling biofilters and rotating biological contactors (SIDX 32–36), were highlighted as impractical for postlagoon use due to their tendency to clog and inability to maintain sufficient biofilm surface area for nitrification under low-loading conditions.^80^

Although these technologies are not currently viable for lagoon-based applications, future improvements could enhance their applicability. Key areas include reducing capital and operating costs, improving process robustness under variable lagoon conditions, and developing more efficient nutrient recovery pathways. For example, advances in low-cost fouling-resistant membranes, regenerable sorbents, stable microbial consortia, and energy-efficient electrochemical systems could make these options more practical for small communities. More details on the specific improvements are provided in the Supporting Information (Section 6).

The use of SIDX reveals key trade-offs between nutrient removal performance and real-world implementation factors. For instance, chemical precipitation scored 82 at full scale due to its effectiveness in phosphorus removal, yet it depends on chemical inputs like alum or ferric chloride, which increase operational costs and generate sludge that requires proper disposal.^72^ Macrophyte floating-bed systems, with a SIDX of 74, are valued for their low cost and minimal maintenance but exhibit diminished performance in colder climates, limiting their year-round reliability.^89^ Upflow denitrification filters, while demonstrating excellent nutrient removal (80% for ammonia, 85% for total nitrogen), received a low SIDX of 35 due to their sensitivity to temperature fluctuations, longer startup periods, and the need for skilled operation, factors that hinder feasibility in small, resource-limited communities.^31^

While SIDX offers a structured approach to comparing technologies, it has inherent limitations. Its scoring can involve subjectivity, and the normalization process may oversimplify or obscure critical context-specific trade-offs. Additionally, SIDX does not fully account for site-specific factors such as local climate, regulatory frameworks, or governance capacity, which can significantly affect a technology’s long-term performance and viability.^52^ Operational challenges, such as maintenance intensity, resource availability, and adaptability to changing conditions, are also not fully captured.

Nevertheless, SIDX presents a valuable starting point for guiding technology selection in small-community lagoon systems. To enhance its practical value, future efforts should focus on refining the weighting of criteria, incorporating tools like Life Cycle Assessment (LCA) and Life Cycle Cost Analysis (LCCA), and explicitly integrating local constraints. By addressing these gaps, the SIDX framework can offer even stronger support for sustainable, cost-effective decision-making tailored to the needs of small communities.

## 4 Conclusions

This review offers the most extensive synthesis to date of nutrient management technologies applicable to small-community lagoon wastewater systems, identifying 1,216 documented strategies across five decades. The trajectory of research reflects an evolution from early bench-scale experimentation to growing interest in full-scale applications. This shift is driven by increasing regulatory pressures, as well as advances in system design and process optimization. However, the review also reveals a structural imbalance: much of the literature is shaped by researcher-interest and funding availability, rather than field-validated needs. As a result, some technologies that are widely implemented and effective in real-world lagoon contexts remain underrepresented in publications, while more complex, experimental systems receive disproportionate academic attention.

Biological processes dominate the nutrient removal landscape, offering cost-effective, energy-efficient treatment aligned with the operational realities of small communities. Technologies such as partial-mix aeration, constructed wetlands, and suspended growth systems consistently demonstrate strong field performance. Yet, nutrient removal performance across technologies varies widely due to differences in configuration, environmental sensitivity, and operational oversight, challenges particularly acute in resource-limited settings.

To support practical decision-making, this review introduces the Suitability Index (SIDX), a multicriteria framework that integrates nutrient removal efficiency, technical maturity, environmental sensitivity, and operational feasibility. Results show that SIDX scores increase with development stage, reflecting improved validation and field readiness at scale. Technologies were further categorized as applicable, promising, or not currently viable, with key trade-offs identified between performance potential and implementation constraints. While not a replacement for site-specific assessments, SIDX offers a valuable screening tool to help resource-limited communities prioritize feasible strategies.

Importantly, the review highlights the absence of standardized classification frameworks tailored to lagoon systems, noting that many technologies, particularly modular add-ons and hybrid processes, do not align with conventional typologies, complicating comparison and selection. It also identifies a growing shift toward circularity and resource recovery, with emerging approaches such as algal systems and anaerobic filters offering pathways to transform waste into valuable products. This evolution aligns nutrient management with climate goals and economic resilience but also introduces new challenges, including regulatory uncertainty, market barriers, and operational complexity, especially in small, decentralized settings.

Moving forward, there is strong potential to enhance the SIDX framework by integrating local context, climate resilience, and governance capacity. Incorporating tools such as Life Cycle Assessment (LCA) and Life Cycle Cost Analysis (LCCA) to better quantify emissions, costs, and trade-offs, alongside validating technologies across diverse real-world conditions, will improve the framework’s practical relevance. Embedding circularity metrics and enabling adaptive permitting or incentive-based mechanisms, such as nutrient trading, can further support sustainable, cost-effective solutions, empowering small communities to implement resilient, future-ready nutrient management strategies.

## Supplementary Material





## Notes

Methods and calculation procedures for SIDX (PDF)
Categorized synthesis of over 1000 peer-reviewed studies and detailed scoring criteria; and comprehensive reference tables summarizing key findings (XLSX)
