# Supplementary material for: Toward Sustainable Lagoon Wastewater Treatment: A Review of Nutrient Management Technologies and Their Suitability for Small Communities
Source: ACS ES T Water. 2025 Oct 2;5(11):6200–16. doi: 10.1021/acsestwater.5c00757 (PMC12624740; doi:10.1021/acsestwater.5c00757)
Supplement: Supplementary file 1 [file ew5c00757_si_001.pdf]

Supplementary Information

Table of Contents

1 Introduction 2

1.1 Lagoon wastewater systems in the U.S. 2

1.2 Need for Nutrient Management 2

1.3 Development of Nutrient Management Technologies 3

2 Methods 4

3 Suitability Index (SIDX) 4

3.1 Data Preparation and Scoring 4

3.2 Scoring Rationale and Relevance to Small Communities 4

3.2.1 Integration with Existing Infrastructure 5

3.2.2 Process Complexity 5

3.2.3 Automation Potential 5

3.2.4 Market Availability 5

3.2.5 Sensitivity to Site Factors 5

3.2.6 Dependence on Consumables 5

3.2.7 Process Predictability 6

3.2.8 Maintenance Requirements 6

3.2.9 Nutrient Removal Capacity 6

3.3 Normalization of Scores 6

3.4 Analytic Hierarchy Process (AHP) Pairwise Comparison Matrix 7

4. Technology Classification & Suitability for Small Communities 3

5. SIDX Results 4

5.1 Limitations of Suitability Index 5

6. Improvements for Non-viable Technologies 6

References 7

1 Introduction

1.1 Lagoon wastewater systems in the U.S.

Currently, there are over 8,000 lagoon systems (both discharging and non-discharging to surface water) in operation across the U.S. [1]. Most lagoons are in the Midwest and are prevalent in small and rural communities, typically with populations under 10,000 (Figure S1).

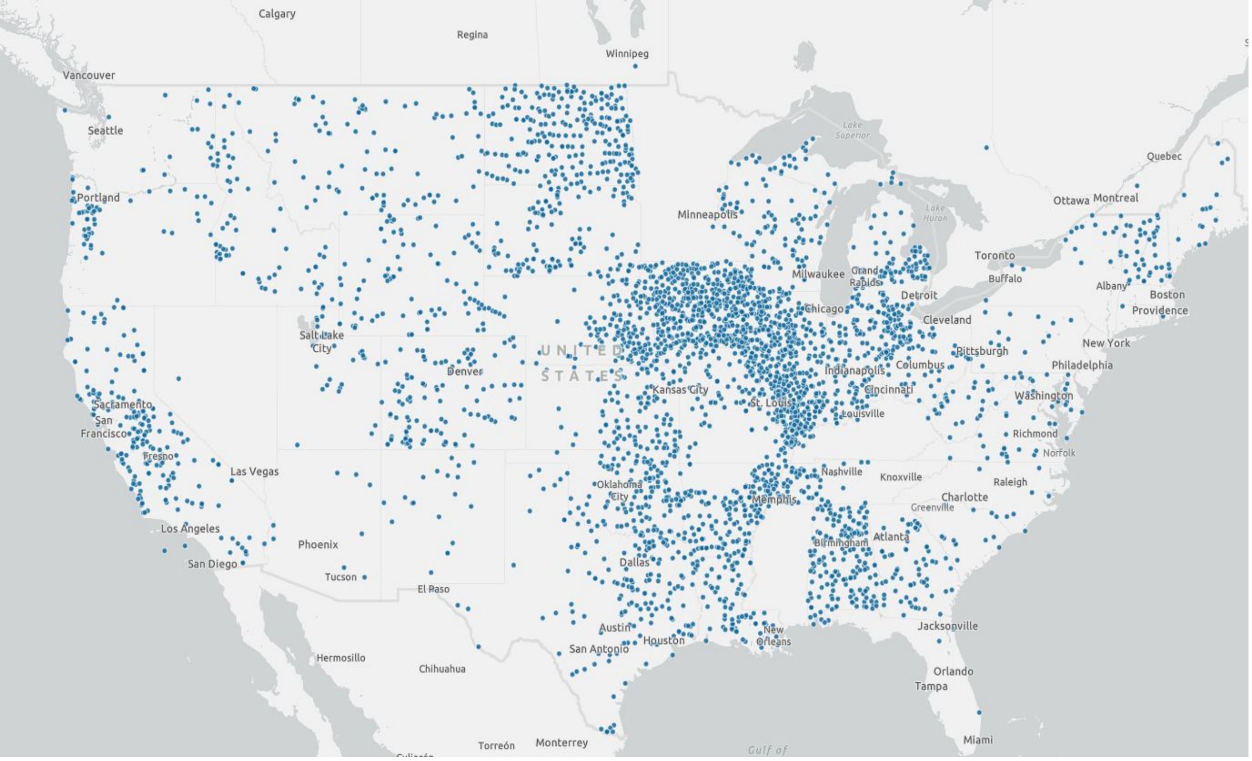

***Figure S1**: Spatial distribution of discharging lagoon wastewater systems serving communities with less than 10,000 inhabitants across the contiguous 48 states in the U.S.*

Discharging lagoons release treated effluent into surface waters and must meet strict nutrient limits for nitrogen and phosphorus, often requiring additional treatment steps like aeration or filtration. In contrast, non-discharging lagoons rely on evaporation, seepage, or land application to manage effluent, and typically face less stringent nutrient regulations. While discharging systems focus on removing nutrients to prevent waterway pollution, non-discharging systems aim to retain or recycle nutrients, often through soil or crop uptake. However, without proper management, non-discharging lagoons risk nutrient buildup in soils or contamination of groundwater. This study focuses specifically on enhancing nutrient management strategies for discharging lagoon systems [2].

1.2 Need for Nutrient Management

In wastewater treatment, nutrients primarily refer to nitrogen and phosphorus compounds, which are essential for the growth of plants and microorganisms[3]. Nitrogen in wastewater exists in several forms. Ammonia (NH_4^+) is one of the most common nitrogen compounds, originating from the breakdown of organic matter and urea in human waste. Nitrate (NO_3^-), another nitrogen

compound, is formed during the nitrification process in treatment systems [4]. Nitrite (NO_2^-), an intermediate product in the conversion of ammonia to nitrate, is also harmful to aquatic ecosystems at elevated levels [5]. Additionally, organic nitrogen, found in proteins and other organic materials, contributes to the total nitrogen load in wastewater [6]. Phosphorus, another key nutrient, is commonly present in wastewater in both organic and inorganic forms [7,8]. Orthophosphates (PO_4^{3-}), the most bioavailable form, often originate from detergents, cleaning products, and organic waste [9]. Polyphosphates, used in industrial and household cleaning agents, break down into orthophosphates over time [8,10]. Organic phosphorus, derived from biological material such as food residues, adds to the total phosphorus load [8].

While nutrients can be beneficial in small quantities, their excessive presence in untreated wastewater can cause significant environmental and public health issues [11]. Ammonia is highly toxic to aquatic life and contributes to oxygen depletion in water bodies [12]. While nitrate is less toxic to aquatic organisms than ammonia, nitrate contamination in drinking water can cause serious health problems, such as methemoglobinemia or "blue baby syndrome" in infants [13]. Excess nitrogen and phosphorus in water bodies can lead to eutrophication, a process that results in excessive growth of algae and aquatic plants. As these organisms decay, they consume oxygen, creating hypoxic or "dead zones" where most aquatic life cannot survive; thus harming aquatic life, and disrupting ecosystems [14,15].

1.3 Development of Nutrient Management Technologies

Bench-scale studies are conducted in controlled laboratory environments to test the fundamental principles of technology. At this stage, the focus is on understanding the chemical, biological, or physical processes involved in nutrient removal while optimizing key operational parameters such as pH, temperature, and chemical dosing [16,17]. However, while these studies provide important foundational knowledge, their findings often lack real-world applicability due to the absence of environmental variability, such as fluctuating wastewater characteristics, seasonal changes, and weather conditions typical of lagoon systems [16].

Pilot-scale studies are done in a scaled-down version of the actual operating environment to simulate real-world lagoon conditions, exposing the technology to variables such as influent variability, temperature fluctuations, and operational stressors [18]. This phase is crucial for assessing the practicality of the technology, evaluating energy and chemical requirements, identifying maintenance challenges, and estimating operational costs [19,20]. Despite providing more reliable performance data than bench-scale studies, pilot-scale systems can be expensive to build and operate, and their findings often require further validation during full-scale implementation [21].

The full-scale phase involves deploying the technology in an operational lagoon system to assess its long-term effectiveness, reliability, and scalability under actual working conditions [22]. Performance data at this stage are the most reliable, as the technology operates under real influent loads, regulatory constraints, and environmental conditions [23]. Full-scale implementation also provides critical insights into long-term operational and maintenance costs, as well as potential environmental trade-offs [24]. However, transitioning from pilot-scale to full-scale often presents a significant financial barrier, particularly for small communities with limited budgets [25]. Many promising technologies fail to reach full-scale deployment due to high capital costs,

insufficient funding, or regulatory uncertainties [26]. Furthermore, technologies must demonstrate compatibility with existing lagoon infrastructure to gain broader market acceptance [27].

2 Methods

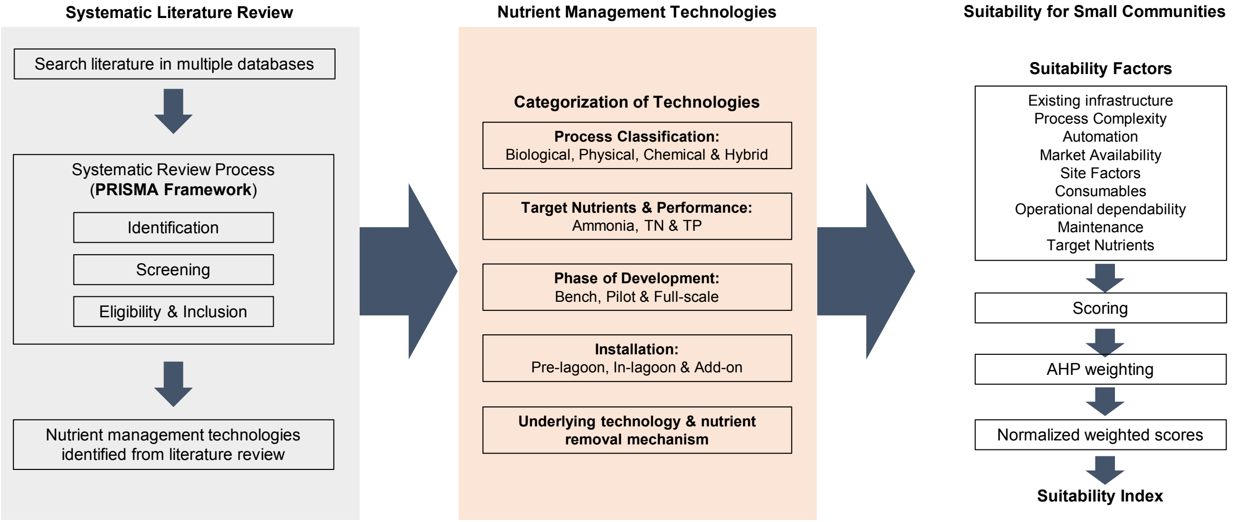

Figure S2: *Overview of the systematic approach used to assess and categorize nutrient management technologies through three key phases: literature review, technology categorization, and suitability evaluation. The Analytic Hierarchy Process (AHP) was applied to quantitatively assess the suitability of each technology for small communities.*

3 Suitability Index (SIDX)

The methodology employed for creating the Suitability Index (SIDX) was guided by the principles of the Analytic Hierarchy Process (AHP) to prioritize and rank nutrient removal technologies for small community lagoon systems. Below is a detailed breakdown of the methods used:

3.1 Data Preparation and Scoring

The process began with the preparation of a dataset containing attributes of various nutrient management technologies. Several criteria were conceptualized to assess the technologies, including existing infrastructure compatibility, process complexity, automation, market availability, site factors, consumables, operational, maintenance requirements, and target nutrients. Each criterion was assigned a scoring mechanism tailored to its nature as outlined in *Supplementary Tables S4* and *S6*.

3.2 Scoring Rationale and Relevance to Small Communities

The Suitability Index (SIDX) scoring system was designed to provide a structured and quantitative framework for evaluating nutrient management technologies in small communities operating lagoon systems. Each criterion was developed to assess key factors influencing feasibility, performance, and long-term sustainability, ensuring that selected technologies align with the unique constraints of resource-limited settings. The following sections explain the rationale behind each question, its relevance to small communities, and how the scoring was applied.

3.2.1 Integration with Existing Infrastructure

One critical consideration is whether a technology can be implemented within an existing lagoon system without requiring significant retrofitting. Small communities often operate within tight budget constraints, making it essential to minimize capital costs by leveraging current infrastructure. Technologies classified as "in-lagoon" require minimal additional infrastructure and are therefore given a score of 1, whereas those requiring pre-lagoon or add-on installations score 0 due to their higher implementation complexity and cost.

3.2.2 Process Complexity

The operational complexity of a technology directly affects its feasibility in small communities with limited technical expertise and operator availability. Simpler technologies are easier to manage and maintain, reducing the likelihood of operational failures. To measure complexity, the number of nutrient removal mechanisms within a technology is used as a proxy. Technologies relying on a single mechanism score 1 for their ease of use, while those with multiple mechanisms score 0, reflecting increased operational and training demands.

3.2.3 Automation Potential

Automation can significantly reduce the need for manual intervention, ensuring consistent system performance and minimizing operational challenges, particularly in remote or underserved areas. The ability to automate a process is determined based on its classification. Chemical, physical, and hybrid systems score 1, as they can be readily automated, while biological processes score 0 due to their reliance on continuous monitoring and operator oversight.

3.2.4 Market Availability

The ease of adoption for a technology depends largely on its market availability. Technologies that are commercially available can be implemented more efficiently, reducing delays and uncertainties. The development phase serves as a proxy for availability: full-scale technologies, which are ready for deployment, score the highest (3), followed by pilot-scale technologies (2), and bench-scale technologies (1), which are still in experimental or developmental stages.

3.2.5 Sensitivity to Site Factors

Environmental conditions, particularly cold temperatures, can impact the performance of certain nutrient removal technologies. In regions where temperatures drop below 5°C, biological activity in lagoon systems may decline, reducing treatment efficiency. To account for this, the presence of biological nutrient removal (BNR) is used as an indicator. Non-BNR systems, which are less affected by temperature variations, score 1, while BNR-based systems, which are more vulnerable to cold conditions, score 0.

3.2.6 Dependence on Consumables

Some technologies require recurring inputs such as chemicals or replacement parts, increasing long-term operational costs. Given that resource availability can be limited in small communities,

lower dependency on consumables is preferred. Technologies that do not rely on chemicals score 1, whereas chemical-based processes, which require regular material inputs, score 0.

3.2.7 Process Predictability

Reliability is essential for wastewater treatment technologies, especially in communities that depend on consistent performance. Predictable systems operate with well-defined processes and outcomes, making them more dependable under variable conditions. To measure predictability, process classification is used: non-biological processes score 1 due to their stable and consistent operation, while biological processes score 0, as they are more susceptible to fluctuations in microbial activity and environmental conditions.

3.2.8 Maintenance Requirements

Long-term feasibility depends on the ease of maintaining a technology, particularly in communities with limited technical resources. Low-maintenance systems are preferable, as they reduce operational burdens and long-term costs. Maintenance needs are assessed based on process classification: biological processes, which typically have fewer moving parts and require less frequent maintenance, score 1, while other systems score 0 due to their higher maintenance demands.

3.2.9 Nutrient Removal Capacity

The ability of a technology to remove multiple nutrients is critical for meeting regulatory and environmental goals. More comprehensive nutrient removal provides greater flexibility and value to small communities. Technologies that remove three key nutrients (ammonia, total nitrogen, and total phosphorus) score the highest (4), followed by those that remove two nutrients (3 for TP/TN, 2 for TP/Ammonia), while technologies that target only ammonia score the lowest (1).

By integrating these criteria into the SIDX framework, small communities can systematically assess nutrient management technologies based on their practical suitability, ensuring alignment with financial, technical, and environmental constraints. The scoring system converts each suitability factor into a measurable attribute, enabling a standardized and objective evaluation of technologies. This approach prioritizes key concerns for small communities, such as cost-effectiveness, operational simplicity, and long-term sustainability, while also considering broader factors like automation and environmental adaptability. By quantifying these elements, the suitability index offers a clear, actionable framework for selecting nutrient management solutions that best fit the unique requirements of lagoon-based wastewater treatment systems.

3.3 Normalization of Scores

Normalization of scores was a critical step in developing the suitability index, ensuring consistency and fairness when comparing technologies across multiple criteria. Each criterion was initially scored using specific scales based on proxies from the dataset, such as binary scoring for "existing infrastructure" (0 or 1) and a multi-level scale for "market availability" (1 to 3). To address these disparities, raw scores were normalized using a linear scaling method. This involved dividing each raw score by the maximum possible score for that criterion, scaling all values to a range between 0 and 1, where 1 represented the highest performance. This approach

ensured all criteria were comparable on a standardized scale, eliminating distortions from differing original scoring formats. The normalized scores were then integrated into the weighted framework derived from the Analytic Hierarchy Process (AHP), reflecting the relative importance of each criterion.

3.4 Analytic Hierarchy Process (AHP) Pairwise Comparison Matrix

The Analytic Hierarchy Process (AHP) offers a structured approach to evaluating nutrient management technologies by systematically comparing criteria based on their relative importance [28]. Using Saaty's 1-9 scale, the AHP Pairwise Comparison Matrix ranks these criteria, enabling decision-makers to prioritize key factors. A score of 1 signifies equal importance between two criteria, while values up to 9 indicate increasing dominance of one over the other. Reciprocal values (1/X) ensure logical consistency, automatically adjusting reverse comparisons [29,30]. This method provides an objective framework for weighing multiple considerations, ensuring that selected technologies align with practical implementation needs and long-term sustainability goals. Accordingly, a pairwise comparison matrix was constructed, as detailed in *Table S1* to determine the relative importance of each criterion.

Table S1: AHP Pairwise Comparison Matrix

Criteria	Existing infrastructure	Process complexity	Automation	Market Availability	Site Factors	Consumables	Operational dependability	Maintenance	Target nutrient
Existing infrastructure	1	3	4	5	5	6	6	5	3
Process complexity	1/3	1	1/5	1/3	1/3	1/3	1/4	1/2	3
Automation	1/4	5	1	1/3	1/4	1/5	1/3	6	2
Market Availability	1/5	3	3	1	1/2	1/3	1/4	5	5
Cold Climate	1/5	3	4	2	1	1/3	1/4	5	6
Consumables	1/6	3	5	3	3	1	1/3	4	5
Operational dependability	1/6	4	3	4	4	3	1	3	4
Maintenance	1/5	2	1/6	1/5	1/5	1/4	1/3	1	2
Target nutrient	1/3	1/3	1/2	1/5	1/6	1/5	1/4	1/2	1

Existing infrastructure was identified as one of the critical factors in the decision-making process. Leveraging existing infrastructure minimizes costs and facilitates easier integration of new technologies. This criterion was rated significantly more important than others, such as *operational dependability*, with a weight of 6, reflecting its foundational role in enabling new systems. Compared to *process complexity* and *automation*, it was considered moderately more critical (weight of 3 and 4, respectively), as these operational aspects depend on having robust infrastructure in place.

Process complexity is another important consideration, particularly for small communities with limited technical expertise. Simpler processes were considered easier to operate and maintain. While *process complexity* was considered more critical than the target nutrient (weight of 3), it was rated less critical than existing infrastructure, with a weight of 1/3, and less significant than *Automation* (1/5). This reflects the understanding that operational simplicity is important but secondary to infrastructure integration and automation factors.

Automation was assumed to hold considerable weight in the matrix as it reduces human intervention, ensures reliability, and promotes consistency, especially in remote or underserved areas. Thus, it was rated more critical than *process complexity* and *maintenance*, with weights of 5 and 6, respectively, as it mitigates operational challenges caused by human error. However, it was rated slightly less important than m*arket availability* (1/3), reflecting that availability may limit the adoption of automated systems.

Market availability plays a vital role in determining the feasibility of technology implementation. If a technology is readily available, it can be adopted more quickly and cost-effectively. It was rated slightly less important than *existing infrastructure* (1/5) but more important than *maintenance* (5), as accessibility to solutions is an immediate enabler. However, its importance is slightly outweighed by *site factors* (1/2), given the operational challenges that might arise in cold climates.

Site factors are particularly important considerations in regions where low temperatures affect biological activity in lagoon systems. Technologies must be adaptable to these conditions to ensure effective performance. This criterion was rated less critical than foundational aspects like *existing infrastructure* (1/5) and operational enablers like *consumables* (1/3). However, it was considered moderately more important than *process complexity* (3) and *automation (4)*, as adapting to environmental challenges directly impacts operational processes.

Consumables, such as chemicals or replacement parts, influence ongoing operational costs and affordability. This factor was rated less critical *than existing infrastructure* or *operational dependability* (1/6 and 1/3, respectively) but more important than *automation* (5), as consumables have a direct impact on long-term costs and resource requirements.

Operational dependability refers to consistent performance under varying conditions, which is essential for small communities relying on lagoon systems. While it was rated less important than *existing infrastructure* (1/6), it was considered to hold more significance than *maintenance* (3), as dependability directly influences the overall effectiveness of the system.

Maintenance is a key factor for the long-term sustainability of lagoon technologies, particularly in communities with limited technical resources. It was rated less critical than *consumables* (1/4)

and *operational dependability* (1/3), as maintenance is often dependent on process simplicity and the availability of consumables. However, it was considered moderately more important than *target nutrients* (2), as ease of maintenance ensures the technology remains viable over time.

Lastly, *target nutrients* focus on the ability of a technology to remove specific nutrients like nitrogen or phosphorus. While essential for meeting regulatory and environmental goals, it was rated the least critical criterion in the matrix. This was based on the understanding that nutrient targeting is a downstream goal that often depends on the success of other foundational and operational factors. It was considered slightly more important than *maintenance* (1/2) due to its role in achieving specific environmental outcomes.

3.5 Consistency in AHP

In AHP, the consistency ratio (CR) serves as an important measure to evaluate the logical coherence of judgments made during pairwise comparisons of criteria. A CR value of 0.10 (or 10%) or less is generally considered acceptable [28]. This threshold indicates that the comparisons are sufficiently consistent, and the results can be trusted for decision-making. When the CR exceeds 0.10, it suggests inconsistency in the judgments, requiring a review and revision of the pairwise comparisons to ensure logical alignment.

The CR was calculated by dividing the consistency index (CI) by the random index (RI), a standard value derived from the average consistency of randomly generated matrices of the same size as described in Equation 1 [28,31].

$$CR = CI/RI \quad (1)$$

The CI itself was calculated using the principle eigenvalue (λ_{max}) of the pairwise comparison matrix (average of the weighted sum vector divided by the weight score) and the number of criteria (n) as described in Equation 2 [28,31].

$$CI = \frac{\lambda_{max} - n}{n-1} \quad (2)$$

The RI values vary based on the size of the matrix (number of criteria), with larger matrices allowing for slightly more inconsistency due to the complexity of judgments. In this study, for a matrix with 9 criteria, the RI is 1.4537 [28]. *Supplementary Table S5: AHP Matrix* provides additional details and final values of the weight score, CI and CR.

3.6 Suitability Index (SIDX) Calculation

Using the AHP-derived weights, the normalized scores for each criterion were combined into a weighted sum to calculate the Suitability Index (SIDX) for each technology, as described in Equations 3 and 4 [28,31].

$$Weighted\ Score\ Aggregate\ (WSA) = \sum_{i=1}^{n=9} (Normalized\ Score_i \times Weight_i) \quad (3)$$

$$SIDX = \ Normalized\ WSA \tag{4}$$

This approach quantified the relative importance of each criterion, allowing higher-weighted factors, such as "existing infrastructure" and "automation," to exert greater influence on the final SIDX compared to lower-weighted factors like "maintenance" or "site factors." The weighted scores for each technology were aggregated to produce the final Weighted Score Aggregate (WSA), which was then normalized to enable easy interpretation and ranking, ultimately generating the SIDX (*Supplementary Table S6: Suitability Index)*.

The SIDX values were verified for consistency, and the dataset was ranked from the most to the least suitable technologies based on the normalized SI scores. The top-ranking technologies were identified and exported for further analysis. This approach allowed for a comprehensive and systematic evaluation of nutrient management technologies, ensuring that the index reflects not only performance and cost but also operational, environmental, and logistical considerations.

4. Technology Classification & Suitability for Small Communities

A key challenge encountered during the review was the sheer number and diversity of nutrient management technologies, often described using inconsistent or overlapping terminology. This lack of standardized classification created difficulties in comparing systems across studies and posed a barrier to interpretation—particularly for small communities with limited technical capacity seeking practical, actionable guidance. To overcome this challenge, the review organized 1,216 instances of nutrient management strategies into 22 distinct underlying technologies, minimizing variability introduced by commercial branding and consolidating approaches that share similar treatment principles. (Figure S2).

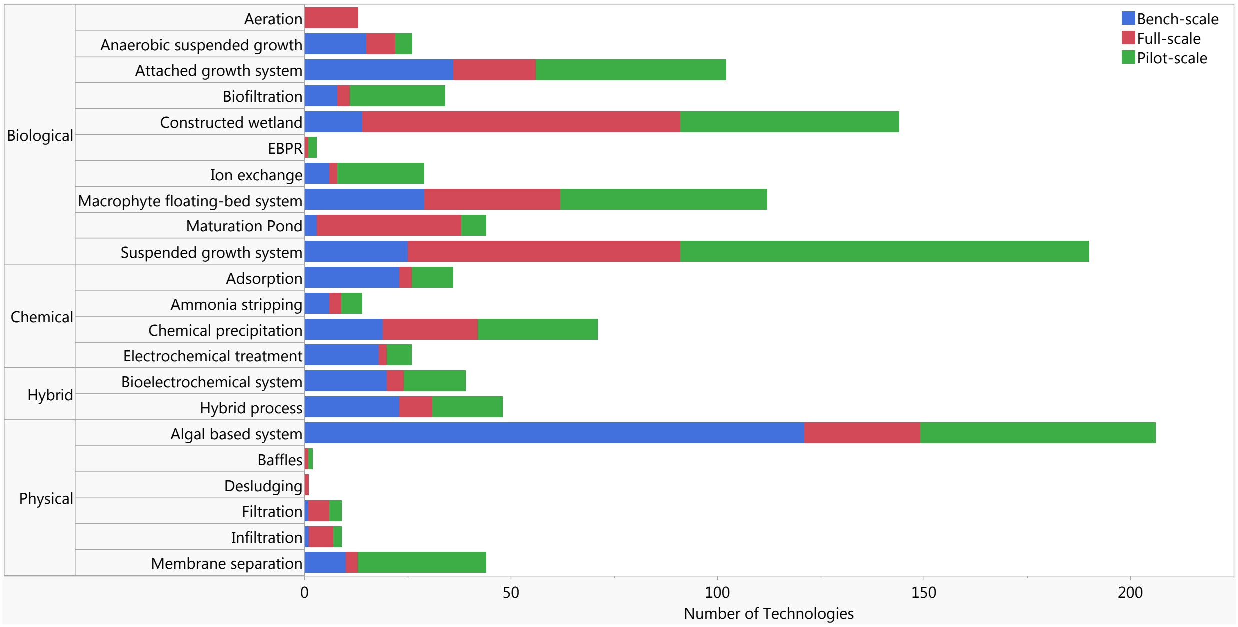

Figure S2: Distribution of nutrient management strategies categorized into 22 distinct types of underlying technologies.

Suspended growth systems and constructed wetlands dominate across pilot and full scales. reflecting their adaptability and maturity for lagoon applications. Algal-based systems, while frequent, was observed to be predominantly studied in bench-scale, showing their lack in market availability.

The underlying technologies were further organized into four overarching categories: (1) conventional systems: proven, widely adopted methods with established implementation frameworks; (2) enhanced biological systems: combine natural processes with engineered components to improve nutrient removal; (3) advanced systems: offer high-performance treatment through specialized designs or greater operational complexity; and (4) hydraulic improvements: optimize lagoon function by enhancing flow patterns and solids management (Figure S3).

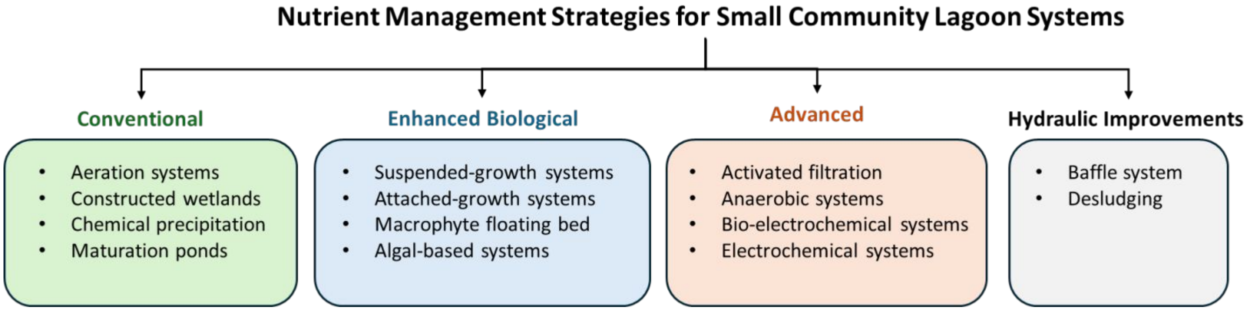

Figure S3. *Summary of nutrient management strategies with select examples that are applicable to small community lagoon wastewater systems.*

5. SIDX Results

A comparison of SIDX scores (0-1scale) across development stages reveals a clear trend: technologies tend to become more suitable as they progress from bench-scale to pilot- and full-scale deployment (Figure S4). At the bench scale, the mean SIDX was 0.077, with a median of 0.057, indicating limited readiness for application in small communities due to the experimental nature of many early-stage systems and the need for further optimization [26]. Pilot-scale technologies showed modest improvement (mean: 0.088, median: 0.069), reflecting increased feasibility following process refinement and partial field validation [32]. The maximum SIDX increased from 0.159 at bench scale to 0.171 at pilot, peaking at 0.236 at full scale, reinforcing the observation that technology suitability improves with development [33]. Focusing on full-scale implementations, SIDX ranged from 0.067 to 0.236 (mean: 0.099, standard deviation: 0.037), indicating moderate suitability, albeit with significant variability. The upper quartile (SIDX > 0.119) represent the most promising solutions such as algal-based and macrophyte floating-bed systems which combine high nutrient removal efficiency with operational reliability and adaptability [34,35].

This variability in SIDX scores arises from multiple factors, including the breadth of classifications within technology groups and context-specific performance. For instance, constructed wetlands comprise two major subtypes—free water surface (FWS) and subsurface flow (SSF) systems—which

differ in hydraulic design, oxygen transfer, and nutrient removal mechanisms. FWS wetlands are generally simpler to build and effective for phosphorus uptake in warmer climates, whereas SSF systems are more compact and better suited for nitrogen removal [36].

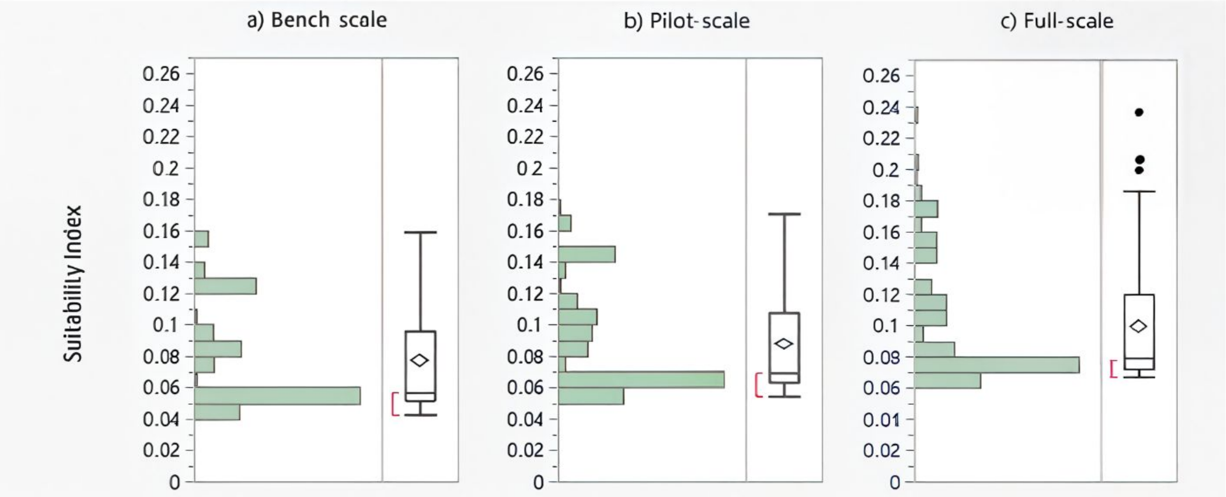

Mean	0.0771	0.0876	0.0991
Std Dev	0.0327	0.0322	0.0370
Std Err Mean	0.0017	0.0015	0.0020
Upper 95% Mean	0.0804	0.0905	0.1031
Lower 95% Mean	0.0739	0.0847	0.0953
N	391	480	342

Figure S4. *Suitability Index (SIDX) distribution by development scale: (a) bench, (b) pilot, and (c) full-scale. Boxplots show the mean (◊) and 95% confidence interval (red bar).*

5.1 Limitations of Suitability Index

The SIDX is a valuable tool for assessing and ranking nutrient removal technologies for lagoon systems, but it has several limitations that warrant careful consideration. One key challenge is the subjectivity inherent in scoring and weighting criteria, as determined through the Analytic Hierarchy Process (AHP). Intangible factors like process complexity and operational dependability are particularly difficult to quantify, which can lead to oversimplifications and reduce the reliability of the results. Additionally, differences in expertise, regional contexts, and organizational priorities can introduce biases, influencing how scores and weights are assigned. While AHP provides a structured and transparent framework, the relative importance assigned to criteria often reflects subjective judgments that may not align with the unique priorities of all small communities. For instance, communities with specific environmental challenges or limited financial resources may emphasize different criteria, limiting the index's universal applicability.

Moreover, the SI does not fully account for dynamic and context-specific factors, such as local regulations, climate and stakeholder preferences which significantly influence technology adoption. These critical considerations are difficult to quantify and incorporate into a standardized framework. Additionally, the index's reliance on normalization to compare diverse criteria can

obscure important trade-offs, such as those between high nutrient removal efficiency and operational costs, which are pivotal for decision-makers in resource-limited settings.

Considering these limitations, the Suitability Index should be viewed as a complementary tool rather than a definitive solution. To improve its effectiveness, site-specific evaluations, robust stakeholder involvement, and transparency in the scoring and weighting processes are essential to ensure the tool aligns with the diverse needs and priorities of small communities.

6. Improvements for Non-viable Technologies

Several nutrient management technologies were identified as “not currently viable” for small community lagoons. Nonetheless, targeted advances could substantially improve their future applicability. Microbial fuel cells and microbial electrolysis cells, for instance, would require more durable electrode materials, greater biofilm stability, and higher power density to achieve cost-effectiveness at scale [37]. Membrane-based processes could become feasible with the development of fouling-resistant and low-cost membranes, paired with simplified cleaning and maintenance protocols[38]. Ion exchange and adsorption systems would benefit from significantly affordable and regenerable sorbents that minimize chemical use and waste disposal challenges; however, their effectiveness is often maximized under higher-strength influent conditions than those typically found in lagoons, which may limit applicability unless concentration steps or selective sorbents are developed [39,40].

Biological processes such as Enhanced Biological Phosphorus Removal (EBPR) and Anammox could become more suitable with modifications that address their sensitivity to dilute influent, biomass washout, and variable operating conditions. Conventional lagoons generally lack the readily biodegradable carbon, controlled redox environments, and stable sludge ages required for these processes to function reliably[24]. However, their applicability could be improved through the use of hybrid configurations such as baffled or compartmentalized cells to create predictable anaerobic–aerobic zones, the addition of attached-growth to enhance biomass retention, and side-stream fermenters to generate volatile fatty acids for EBPR [6,10]. For Anammox, intermittent aeration, low-DO control, and temperature stabilization measures would be critical to maintaining stable microbial communities and partial nitritation [41,42].

Electrochemical approaches such as electrolysis and electrodialysis could become more applicable to municipal lagoons if advances are made in energy efficiency, electrode durability, and overall process resilience under the dilute conditions characteristic of lagoon influent. At present, their performance is constrained by high energy requirements and sensitivity to fouling, but coupling these processes with renewable energy sources and developing longer-lasting, low-maintenance electrode materials would help reduce operating costs and improve reliability [43,44].

Overall, reducing capital and operating costs, enhancing operational stability under lagoon-specific conditions, and establishing efficient nutrient recovery pathways are the most critical improvements needed to shift these technologies from being non-viable toward practical adoption in small-community lagoon systems.

References

1. U.S. EPA. The Universe of Lagoons: An analysis of state and tribal lagoon wastewater treatment systems and socioeconomic, environmental justice, and compliance patterns in small, rural communities in the United States. Washington, D.C.: U.S. Environmental Protection Agency; 2022 May. Report No.: EPA-823-R-22-001.

2. U.S. EPA. Principles of Design and Operations of Wastewater Treatment Pond Systems for Plant Operators, Engineers, and Managers. Cincinnati, OH: U.S. Environmental Protection Agency.; 2011 Aug. Report No.: EPA/600/R-11/088.

3. Boyden BH, Rababah AA. Recycling nutrients from municipal wastewater. Desalination. 1996;106(1–3):241–6.

4. Bhattacharya R, Mazumder D. Simultaneous nitrification and denitrification in moving bed bioreactor and other biological systems. Bioprocess Biosyst Eng. 2021 Apr 1;44(4):635–52.

5. Tchobanoglous G, Burton F, Stensel HD. Wastewater engineering: Treatment and reuse. Am Water Works Assoc J. 2003 May;95(5):201.

6. Archer HE, O’Brien BM. Improving nitrogen reduction in waste stabilisation ponds. Water Sci Technol. 2005;51(12):133–8.

7. Barnard JL, Dunlap P, Steichen M. Rethinking the Mechanisms of Biological Phosphorus Removal. Water Environ Res Res Publ Water Environ Fed. 2017 Nov 1;89(11):2043–54.

8. Carrillo V, Fuentes B, Gómez G, Vidal G. Characterization and recovery of phosphorus from wastewater by combined technologies. Rev Environ Sci Biotechnol. 2020 June;19(2):389–418.

9. Bowers KE, Westerman PW. Performance of cone-shaped fluidized bed struvite crystallizers in removing phosphorus from wastewater. Trans Am Soc Agric Eng. 2005;48(3):1227–34.

10. Bunce JT, Ndam E, Ofiteru ID, Moore A, Graham DW. A Review of Phosphorus Removal Technologies and Their Applicability to Small-Scale Domestic Wastewater Treatment Systems. Front Environ Sci. 2018 Feb 22;6:8.

11. U.S. EPA. Life Cycle and Cost Assessments of Nutrient Removal Technologies in Wastewater Treatment Plants. U.S. Environmental Protection Agency; 2021 Aug. Report No.: EPA 832-R-21-006.

12. Camargo Valero MA, Read LF, Mara DD, Newton RJ, Curtis TP, Davenport RJ. Nitrification–denitrification in waste stabilisation ponds: a mechanism for permanent nitrogen removal in maturation ponds. Water Sci Technol. 2010 Mar 1;61(5):1137–46.

13. Shin HK, Polprasert C. Ammonia nitrogen removal in attached-growth ponds. J Environ Eng. 1988;114(4):846–63.

14. Ajie GS, Prihatiningtyas E. Nutrients removal from integrated multi-trophic aquaculture (IMTA) water using waste stabilization ponds (WSP). In: 2nd International Conference on

Tropical Wetland Biodiversity and Conservation, 23-24 Oct 2021. UK: IOP Publishing; 2022. p. 012029 (7 pp.). (IOP Conf. Ser., Earth Environ. Sci. (UK); vol. 976).

15. Ayaz SÇ, Aktaş Ö, Fındık N, Akça L, Kınacı C. Effect of recirculation on nitrogen removal in a hybrid constructed wetland system. Ecol Eng. 2012 Mar;40:1–5.

16. Mburu N. Experimental and Modeling Studies of Horizontal Subsurface Flow Constructed Wetlands Treating Domestic Wastewater. ProQuest LLC; 2013.

17. McLean BM, Baskaran K, Connor MA. The use of algal-bacterial biofilms to enhance nitrification rates in lagoons: Experience under laboratory and pilot-scale conditions. 4th Int Spec Conf Waste Stab Ponds Technol Environ April 20 1999 - April 23 1999. 2000;42(10–11):187–94.

18. Belmont MA, Cantellano E, Thompson S, Williamson M, Sanchez A, Metcalfe CD. Treatment of domestic wastewater in a pilot-scale natural treatment system in central Mexico. Ecol Eng. 2004;23(4–5):299–311.

19. Giesen A, Loosdrecht MV, Pronk M, Robertson S, Thompson A. Aerobic Granular Biomass Technology: recent performance data, lessons learnt and retrofitting conventional treatment infrastructure. Proc Water Environ Fed. 2016 Jan 1;2016(11):1913–23.

20. Pishgar R, Banmann CL, Chu A. Pilot-Scale Investigation of Floating Treatment Wetlands as Retrofits to Waste-Stabilization Ponds for Efficient Domestic Wastewater Treatment. J Environ Eng U S. 2021;147(6).

21. Salzmann RD, Ackerman JN, Cicek N. Pilot-scale, on-site investigation of crushed recycled glass as tertiary filter media for municipal lagoon wastewater treatment. Environ Technol. 2022 Jan 2;43(1):51–9.

22. Papadopoulos FH, Tsihrintzis VA. Assessment of a full-scale duckweed pond system for septage treatment. Environ Technol. 2011;32(7):795–804.

23. Pishgar R, Lee J, Dominic JA, Hosseini S, Tay JH, Chu A. Augmentation of Biogranules for Enhanced Performance of Full-Scale Lagoon-Based Municipal Wastewater Treatment Plants. Appl Biochem Biotechnol. 2020 May;191(1):426–43.

24. Espinosa MF, Von Sperling M, Verbyla ME. Performance evaluation of 388 full-scale waste stabilization pond systems with seven different configurations. Water Sci Technol. 2017;75(4):916–27.

25. Daee M, Gholipour A, Stefanakis AI. Performance of pilot Horizontal Roughing Filter as polishing stage of waste stabilization ponds in developing regions and modelling verification. Ecol Eng. 2019 Nov;138:8–18.

26. Lam KL, Zlatanović L, van der Hoek JP. Life cycle assessment of nutrient recycling from wastewater: A critical review. Water Res. 2020 Apr 15;173:115519.

27. Mayo AW, Abbas M. Removal mechanisms of nitrogen in waste stabilization ponds. Phys Chem Earth. 2014;72:77–82.

28. Russo RDFSM, Camanho R. Criteria in AHP: A Systematic Review of Literature. Procedia Comput Sci. 2015;55:1123–32.

29. Saaty TL. A scaling method for priorities in hierarchical structures. J Math Psychol. 1977 June;15(3):234–81.

30. Saaty TL. How to Make a Decision: The Analytic Hierarchy Process. Interfaces. 1994 Dec;24(6):19–43.

31. Khaira A, Dwivedi RK. A State of the Art Review of Analytical Hierarchy Process. Mater Today Proc. 2018;5(2):4029–35.

32. Rout PR, Shahid MK, Dash RR, Bhunia P, Liu D, Varjani S, et al. Nutrient removal from domestic wastewater: A comprehensive review on conventional and advanced technologies. J Environ Manage. 2021 Oct 15;296:113246.

33. Rosemarin A, Macura B, Carolus J, Barquet K, Ek F, Järnberg L, et al. Circular nutrient solutions for agriculture and wastewater – a review of technologies and practices. Curr Opin Environ Sustain. 2020 Aug 1;45:78–91.

34. Skoyles A, Chaganti SR, Mundle SOC, Weisener CG. "Nitrification kinetics and microbial community dynamics of attached biofilm in wastewater treatment." Water Sci Technol. 2020;81(5):891–905.

35. Zhao W, Vermace RR, Mattes TE, Just C. Impacts of ammonia loading and biofilm age on the prevalence of nitrogen-cycling microorganisms in a full-scale submerged attached-growth reactor. Water Environ Res. 2021;93(5):787–96.

36. Almuktar SAAAN, Abed SN, Scholz M. Wetlands for wastewater treatment and subsequent recycling of treated effluent: a review. Environ Sci Pollut Res. 2018;25(24):23595–623.

37. Angelaalincy MJ, Navanietha Krishnaraj R, Shakambari G, Ashokkumar B, Kathiresan S, Varalakshmi P. Biofilm Engineering Approaches for Improving the Performance of Microbial Fuel Cells and Bioelectrochemical Systems. Front Energy Res [Internet]. 2018 [cited 2024 Jan 16];6. Available from: https://www.frontiersin.org/articles/10.3389/fenrg.2018.00063

38. Jayaraman J, Kumaraswamy J, Rao YKSS, Karthick M, Baskar S, Anish M, et al. Wastewater treatment by algae-based membrane bioreactors: a review of the arrangement of a membrane reactor, physico-chemical properties, advantages and challenges. RSC Adv. 2024;14(47):34769–90.

39. Guida S, Conzelmann L, Remy C, Vale P, Jefferson B, Soares A. Resilience and life cycle assessment of ion exchange process for ammonium removal from municipal wastewater. Sci Total Environ. 2021 Aug;783:146834.

40. Huang X, Guida S, Jefferson B, Soares A. Economic evaluation of ion-exchange processes for nutrient removal and recovery from municipal wastewater. Npj Clean Water. 2020 Mar 18;3(1):7.

41. Guo Q, Xing BS, Li P, Xu JL, Yang CC, Jin RC. Anaerobic ammonium oxidation (anammox) under realistic seasonal temperature variations: Characteristics of biogranules and process performance. Bioresour Technol. 2015 Sept;192:765–73.

42. Cho S, Takahashi Y, Fujii N, Yamada Y, Satoh H, Okabe S. Nitrogen removal performance and microbial community analysis of an anaerobic up-flow granular bed anammox reactor. Chemosphere. 2010 Feb;78(9):1129–35.

43. Ju X, Wu S, Huang X, Zhang Y, Dong R. How the novel integration of electrolysis in tidal flow constructed wetlands intensifies nutrient removal and odor control. Bioresour Technol. 2014 Oct;169:605–13.

44. Vineyard D, Hicks A, Karthikeyan KG, Davidson C, Barak P. Life cycle assessment of electrodialysis for sidestream nitrogen recovery in municipal wastewater treatment. Clean Environ Syst. 2021 June;2:100026.
